# Synthesis, biophysical properties, and RNase H activity of 6’-difluoro[4.3.0]bicyclo-DNA

**DOI:** 10.3762/bjoc.15.9

**Published:** 2019-01-08

**Authors:** Sibylle Frei, Adam K Katolik, Christian J Leumann

**Affiliations:** 1 Department of Chemistry and Biochemistry, University of Bern, Freiestrasse 3, 3012 Bern, Switzerland

**Keywords:** DNA/RNA affinity, fluorinated cyclopropanes, fluorinated nucleic acids, RNase H activity, sugar modified nucleosides

## Abstract

Here we present the synthesis, the biophysical properties, and the RNase H profile of 6’-difluorinated [4.3.0]bicyclo-DNA (6’-diF-bc^4,3^-DNA). The difluorinated thymidine phosphoramidite building block was synthesized starting from an already known *gem*-difluorinated tricyclic glycal. This tricyclic siloxydifluorocyclopropane was converted into the [4.3.0]bicyclic nucleoside via cyclopropane ring-opening through the addition of an electrophilic iodine during the nucleosidation step followed by reduction. The *gem*-difluorinated bicyclic nucleoside was then converted into the corresponding phosphoramidite building block which was incorporated into oligonucleotides. Thermal denaturation experiments of these oligonucleotides hybridized to complementary DNA or RNA disclosed a significant destabilization of both duplex types (Δ*T*_m_/mod = −1.6 to −5.5 °C). However, in the DNA/RNA hybrid the amount of destabilization could be reduced by multiple insertions of the modified unit. In addition, CD spectroscopy of the oligonucleotides hybridized to RNA showed a similar structure than the natural DNA/RNA duplex. Furthermore, since the structural investigation on the nucleoside level by X-ray crystallography and ab initio calculations pointed to a furanose conformation in the southern region, a RNase H cleavage assay was conducted. This experiment revealed that the oligonucleotide containing five modified units was able to elicit the RNase H-mediated cleavage of the complementary RNA strand.

## Introduction

**Figure 1:**
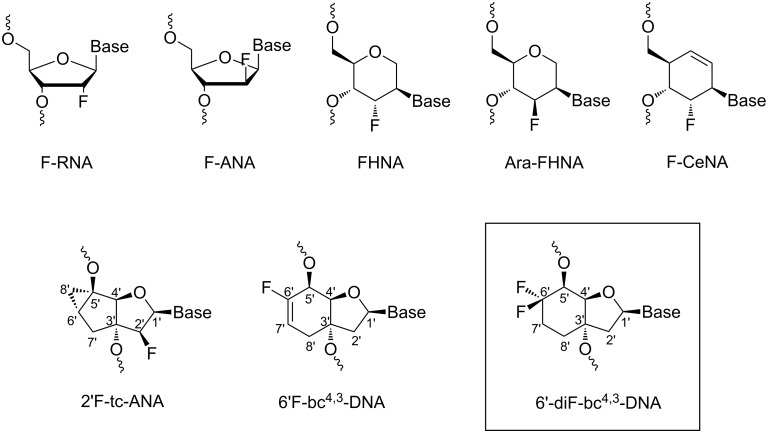
Chemical structure of selected fluorine-modified nucleic acids.

The fluorine atom is a very attractive substituent in medicinal chemistry due to the beneficial biological effects induced by this atom on the overall drug behaviour [1–5]. The positive influences on the drug behaviour is not limited to small molecules but is also valid for antisense oligonucleotides (AONs) [6]. An effective way to tune the properties of antisense oligonucleotides is by the insertion of the fluorine atom in the sugar moiety of the nucleoside. In this way, the sugar pucker can be controlled which ideally results in an increased affinity towards complementary RNA [7]. An improved affinity for RNA as complement can be found in DNA oligonucleotides containing 2’-deoxy-2’-fluoro-RNA (F-RNA) [8] or 2’-deoxy-2’-fluoroarabino nucleic acid (F-ANA, Figure 1) [9]. In the former the sugar pucker adopts a C3’-*endo* conformation [10] and the duplex formation is entropically stabilized. The reason for the stabilization is an increased strength of the Watson–Crick base pairing and base stacking interactions due to the electronic effects of the axially oriented 2’-fluorine atom [11–12]. Additionally, FC–H···O hydrogen bonds between the 2’-fluorine and the 4’-oxygen or 5’-oxygen of the 3’-adjacent nucleotide are thought to favourably contribute in both F-RNA and F-ANA duplexes [13]. Furthermore, in duplexes of the F-ANA with complementary RNA, internucleosidic C–H···F–C pseudohydrogen bonds are proposed at pyrimidine-purine steps to additionally stabilize the structure [14–15]. The β-orientation of the fluorine substituent in F-ANA leads to a *gauche* interaction between O4’–C1’–C2’–F2’ favouring the C2’-*endo*/O4’-*endo* conformations of the sugar in solution [16–17]. These DNA-like sugar conformations cause that F–ANA is among the few modifications which can trigger the cleavage of the RNA strand of an AON/RNA hybrid structure by the endonuclease RNase H [9,18]. Both, the F–ANA and the F–RNA, are appealing modifications for several oligonucleotide-based silencing applications [8,19–25].

Also evaluated on their antisense properties were the 3’-fluorinated hexitol nucleic acids FHNA and Ara-FHNA (Figure 1) with the fluorine in axial or equatorial orientation, respectively. Both modifications preferentially adopt a chair conformation with the nucleobase in axial orientation which mimics the C3’-*endo* conformation of the furanose ring. Thermal denaturation experiments with complementary RNA displayed a duplex stabilization for FHNA and a duplex destabilization for Ara-FHNA. The reason for the stabilization of the former was accounted to a combination of the increased rigidity of the six-membered ring and the positioning of the axial oriented fluorine atom pointing into the minor groove. Conversely, in the Ara-FHNA, repulsive electrostatic interactions between the fluorine atom and the 4’-oxygen of the 3’-adjected nucleotide resulted in a partial unstacking of the nucleobases and a destabilizing effect upon duplex formation [26]. Also other fluorinated nucleic acids such as 2’-fluorocyclohexenyl nucleic acid (F-CeNA, Figure 1) [27] and other modifications [28–31] have been analyzed on their antisense properties.

In our own work we already investigated the effect of the fluorine substituent at various positions of the [3.3.0]bicyclo-DNA (bc-DNA) and tricyclo-DNA (tc-DNA) scaffold. All these modifications unveiled an either identical or slightly increased affinity versus complementary RNA compared to their non-fluorinated compounds [32–35]. Interestingly, the 2’F-tc-ANA (Figure 1) exhibited in a sequence- and composition-dependent manner the ability to induce the RNase H cleavage of the complementary RNA strand [35]. In continuation of our work we became interested in the fluorination of [4.3.0]bicyclo-DNA [36]. Consequently, the 6’-position of the [4.3.0]bicyclo-DNA was substituted with a difluoromethylene group, and the structural effect of this functional unit was explored. Herein we report on the synthesis and properties of the 6’-diF-bc^4,3^-thymidine analog (Figure 1), and the biophysical properties of oligonucleotides containing this modification. Moreover, we investigated the substrate recognition of the 6’-diF-bc^4,3^-T analog by RNase H. In addition, the RNase H experiment was also performed with the previously report 6’F-bc^4,3^-DNA (Figure 1) [37].

## Results and Discussion

### Synthesis of the phosphoramidite building block

**Scheme 1:**
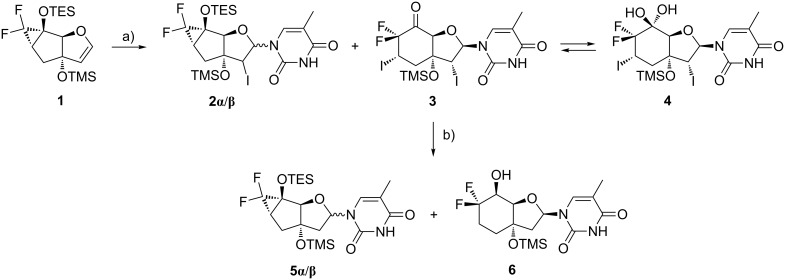
Synthesis of the bicyclic nucleoside **6**. Reagents and conditions: a) BSA, thymine, NIS, DCM, 0 °C to rt, 26 h; b) Bu_3_SnH, AIBN, toluene, 90 °C, 30 min, 34% (**5α/β**), 48% (**6**) over two steps.

In the literature there exist several procedures to construct an α,α-difluoroketone from a corresponding siloxydifluorocyclopropane [38–40]. However, based on previous observations in the nucleoside synthesis of the 6’F-bc^4,3^-T [37] we thought to construct the 6’-diF-bc^4,3^ building block in utilizing this methodology. Along this synthesis, the glycal **1** was treated with *N*-iodosuccinimide (NIS) in the presence of persilylated thymine to produce the iodine intermediates **2α/β** (Scheme 1, Table 1, entry 1). These instable intermediates were then directly reduced with tributyltin hydride (Bu_3_SnH) to yield the tricyclic nucleosides **5α/β** as main compounds. However, we observed the occurrence of the *gem*-difluorinated bicyclic nucleoside **6** as the main side product. Since nucleoside **6** possessed the desired stereochemistry at the 1’- and the 5’-positions, we investigated the mechanism of its formation in more detail to be able to increase its yield. To determine in which of the two steps the formation of the nucleoside **6** took place, they had to be analyzed separately. Therefore, a sample of the iodinated nucleosides **2α/β** was purified and subjected to the reduction reaction, where nucleosides **5α/β** were formed as single products (Table 1, entry 2). Also, the conversion of the nucleosides **5α/β** into the bicyclic derivative **6** could be ruled out (Table 1, entry 3). Consequently, the bicyclic derivative **6** was thought to have its origin in the NIS-mediated nucleosidation step. In analyzing the crude reaction product in more detail, apart from the iodinated nucleosides **2α/β**, the presence of an inseparable mixture of two diiodo-substituted products was unveiled. The major product was the bicyclic *gem*-diol **4** and the minor one the corresponding ketone **3**. We hypothesize that the α,α-difluoroketone **3** was formed through a cyclopropane ring-opening followed by the addition of the electrophilic iodine in accordance to what was reported by the Dilman group [40]. The α,α-difluoroketone **3** rapidly underwent hydration in a reversible way to form the *gem*-diol **4**. The stereochemistry of compound **4** could be assessed indicating the selective formation of the β-nucleoside with the addition of the iodine at the 7’-position from the *exo*-face. Even though we were not able to verify the relative configuration of ketone **3**, we expect the same stereochemistry at the 1’-, 2’- and 7’-positions as in nucleoside **4**. The confirmation that the diiodo-substituted derivatives **3/4** were the precursors of nucleoside **6** came from an experiment in which **3/4** was treated with Bu_3_SnH resulting in nucleoside **6** as the only observed product (Table 1, entry 4). The *S*-configuration at the 5’-position of nucleoside **6** could be explained by a Felkin–Ahn transition state with the hydrogen radical attacking from the less hindered *exo*-face [41–42]. Optimisation of the nucleosidation conditions to an increased amount of NIS (2 equiv) and prolongation of the reaction time in combination with the adjustment of the Bu_3_SnH amount to 3 equivalents led to higher yields of the bicyclic nucleoside **6** (Table 1, entry 5). An additional proof for the reaction mechanism came from the outcome of the reaction where a tricyclic sugar was first treated with NIS and then with Bu_3_SnH resulting as expected in a *gem*-difluorinated bicyclic sugar (Scheme S1, Supporting Information File 1). This reaction also ruled out the involvement of the nucleobase or the iodine at the 2’-position in the reaction mechanism.

**Table 1:** Evaluation of the reaction mechanism for the production of bicyclic nucleoside **6**.

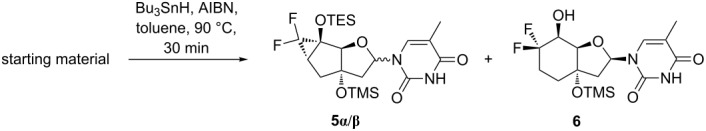					

Entry	Starting material	Bu_3_SnH (equiv)	AIBN (equiv)	Yield **5α/β** [%]	Yield **6** [%]

1	**1**^a^	1.5	0.5	70^b^	21^b^
2	**2α/β**	1.5	0.1	82	–
3	**5α/β**	2.5	0.1	93	–
4	**3/4**	3.1	0.1	–	64
5	**1**^c^	3.5	0.1	34^b^	48^b^

^a^First treated with: thymine (3 equiv), BSA (4.5 equiv), NIS (1.5 equiv), DCM, 0 °C to rt, 4.5 h. ^b^Yield over two steps. ^c^First reacted with: thymine (3 equiv), BSA (4.5 equiv), NIS (2 equiv), DCM, 0 °C to rt, 26 h.

**Scheme 2:**
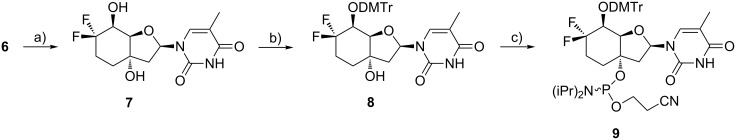
Synthesis of the thymidine phosphoramidite building block **9**. Reagents and conditions: a) HF-pyridine, DCM/pyridine 5:1, 0 °C to rt, 2.5 h, 64%; b) DMTr-OTf, DCM/pyridine 1:2, rt, 22 h, 56%; c) CEP-Cl, DIPEA, THF, rt, 3 h, 73%.

Having attained nucleoside **6**, the synthesis towards the building block for DNA-synthesis continued by subsequent desilylation of this derivative producing intermediate **7** (Scheme 2). DMTr-protection of compound **7** at the 5’-oxygen with in situ prepared DMTr-OTf [43–44] followed by phosphitylation at the 3’-oxygen afforded the phosphoramidite **9**.

### X-ray structure and molecular modeling of the 6’-diF-bc^4,3^ nucleoside

**Figure 2:**
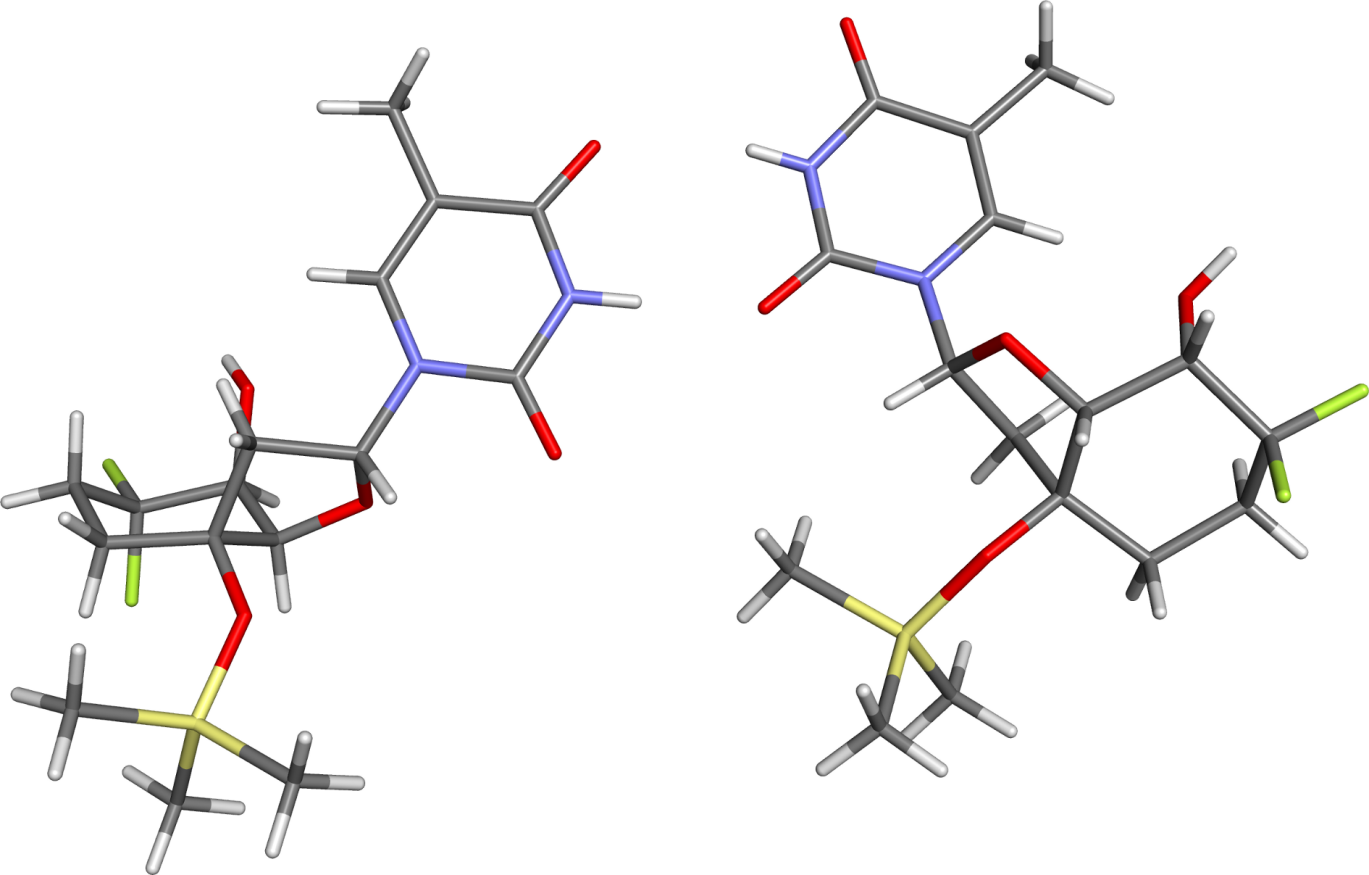
X-ray structure of nucleoside **6a** (left) and **6b** (right).

To verify the relative configuration of nucleoside **6**, crystals of this compound were subjected to X-ray diffraction analysis. The asymmetric unit of a single crystal of nucleoside **6** contained two independent molecules which differed only in the conformation around the C3’–O3’ bond (Figure 2, Table 2, and Tables S1–S3, Supporting Information File 1) [45]. The sugar pucker of both molecules expressed the C2’-*endo* conformation with the pseudorotation phase angle *P* adopting values of 175° (**6a**) and 181° (**6b**), respectively. The maximum puckering amplitude *ν*_max_ was 43° (**6a**) and 40° (**6b**). Furthermore, the nucleobase displayed an *anti* orientation. The carbocyclic ring adopted a chair conformation. As a consequence, the angle γ was aligned in the *synclincal* range and the 5’-hydroxy group in an axial arrangement. Additionally, the distance between the 5’-oxygen and the equatorial fluorine atom F_a_ of 2.80 Å (**6a**) and 2.73 Å (**6b**) correlated with the sum of their van der Waals radii (Table S1, Supporting Information File 1). Interestingly, the two fluorine atoms had an effect on the C5’–C6’ and the C6’–C7’ bond length which were shorter than other C–C bonds in the cyclohexyl ring (Table S2, Supporting Information File 1). Furthermore, the difluoromethylene unit affected the C5’–C6’–C7’ angle and the F–C6’–F angle. The former was widened and the latter shortened compared to the structure of the non-fluorinated bc^4,3^-T (Table 2 and Table S3, Supporting Information File 1). This phenomenon was also observed for other difluoromethylene containing compounds [46]. Apart from that, the observed parameters of the 6’-diF-bc^4,3^-T were very similar to the ones of the bc^4,3^-T, indicating that at least on the nucleoside level the fluorine atoms seemed to have a minor effect on the overall structure.

**Table 2:** Selected parameters from the crystal structure of nucleoside **6** and the standard bc^4,3^-T.

Nucleoside	P [°]	ν_max_ [°]	γ [°]	δ [°]	χ [°]	C5’–C6’–C7’ [°]	X–C6’–X^a^ [°]

**6a**	175	43	74	158	−108	114	105
**6b**	181	40	72	164	−82	114	105
bc^4,3^-T (a)^b^	174	42	70	162	−105	111	108
bc^4,3^-T (b)^b^	166	43	71	154	−120	110	108

^a^**6a/b**: X = F, bc^4,3^-T: X = H. ^b^The structures a and b were two different molecules in the same unit. Data taken from ref [36].

**Figure 3:**
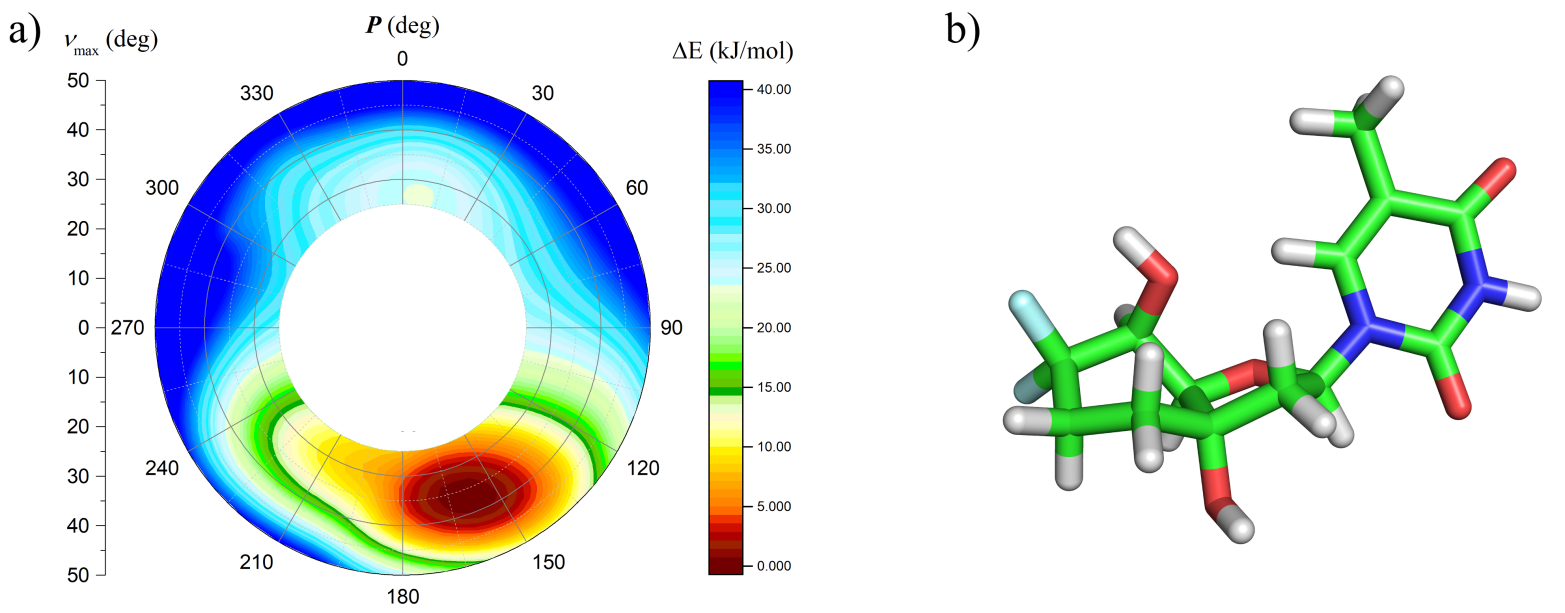
a) Potential energy profile versus pseudorotation phase angle of nucleoside **7** and b) its minimal energy conformer.

To further study the preferred sugar pucker of the 6’-diF-bc^4,3^-T nucleoside, a potential energy profile versus pseudorotation phase angle of nucleoside **7** was calculated using quantum mechanical methods. For the calculations we used the Gaussian 09 software package [47] at the second order Møller–Plesset (MP2) level of theory, the 6-311G* basis set, and the same methodology as for the 6’F-bc^4,3^- T [37]. The obtained energy profile of nucleoside **7** (Figure 3a) surprisingly showed only one single low energy region in the Southern area of the pseudorotational cycle. The minimal energy conformer of nucleoside **7** adopted a C2’-*endo* furanose conformation (*P* = 160°) and a twist-boat orientation of the carbocyclic unit (Figure 3b). Hence, the angle γ took up a *synclincal* arrangement and the 5’-hydroxy group a pseudoaxial orientation. Again, the spacing between the 5’-oxygen and the equatorial aligned fluorine atom F_a_ of 2.61 Å corresponded to the sum of their van der Waals radii. Interestingly, this distance was shorter in the minimal conformer of nucleoside **7** than in the obtained crystal structure of derivative **6** (Table S1, Supporting Information File 1). The reason for that can be attributed to the different conformations of the six-membered rings in these two structures. Apart from that, the two structures were very similar.

### Synthesis of the modified oligonucleotides and their thermal melting profiles

The phosphoramidite building block **9** was incorporated into oligonucleotides and duplexed to complementary DNA and RNA to determine the effect of the 6’-diF-bc^4,3^-T modification on the helical structure and duplex thermostability. The sequences chosen for the investigation were the same as previously used for the 6’F-bc^4,3^-DNA (**ON1**: 5’-d(GGA TGT TCt CGA)-3’, **ON2**: 5’-d(GGA tGT TCT CGA)-3’, **ON3**: 5’-d(GGA TGt tCT CGA)-3’, **ON4**: 5’-d(GCA ttt ttA CCG)-3’) [37]. UV-melting experiments of the modified duplexes were recorded to assess the affinity of the 6’-diF-bc^4,3^-T modified oligonucleotides towards complementary DNA and RNA (Table 3). A single insertion of a 6’-diF-bc^4,3^-T into DNA strands led to a remarkable duplex destabilization versus both complements (Δ*T*_m_/mod = −1.6 to −5.5 °C) in a sequence specific manner but with a slight lesser degree of destabilization towards complementary DNA. However, the degree of destabilization was further minimized with two consecutive insertions of the 6’-diF-bc^4,3^-T (Δ*T*_m_/mod = −4.2 °C and −3.4 °C for complementary DNA and RNA, respectively), whereas five consecutive 6’-diF-bc^4,3^-T units depressed the duplex stability of both structures again (Δ*T*_m_/mod = −5.4 °C and −4.5 °C for complementary DNA and RNA, respectively). Interestingly, the DNA/RNA hybrid structure better accommodated multiple 6’-diF-bc^4,3^-Ts than a single insertion. Additionally, the hybrid structure also better tolerated multiple 6’-diF-bc^4,3^-T units than the DNA/DNA duplex. The reason for the destabilizing nature of the 6’-diF-bc^4,3^-T versus both complements might be found in repulsive electrostatic interactions between the fluorine atoms and the 5’-oxygen. In the case of multiple insertions it might be possible that these interactions might be reduced or maybe compensated by other more favourable effects. The fact, that the two fluorine atoms of the 6’-diF-bc^4,3^-T have a negative influence on the duplex stability was reflected in the higher *T*_m_ values of ONs containing the non-fluorinated bc^4,3^-T unit paired to DNA or RNA (Table 3). However, the *T*_m_ difference between duplexes containing the 6’-diF-bc^4,3^-T or the 6’F-bc^4,3^-T modifications are harder to be interpreted and may reflect different structural preferences between the cyclohexyl to the cylcohexenyl ring. But in general, the duplex instability in the DNA/DNA structures was more pronounced in the 6’-diF-bc^4,3^-T series, whereas to complementary RNA the modified oligonucleotides exhibited a similar degree of destabilization in both series.

**Table 3:** *T*_m_ and Δ*T*_m_/mod data from UV-melting curves (260 nm) of ONs containing 6’-diF-bc^4,3^-T, 6’F-bc^4,3^-T, or bc^4,3^-T residues in the DNA backbone hybridized to complementary DNA and RNA.

Entry	Sequence (5’ → 3’)^a^	*T*_m_ [°C] vs DNA (Δ*T*_m_/mod [°C])			*T*_m_ [°C] vs RNA (Δ*T*_m_/mod [°C])		
	X =	6’-diF-bc^4,3^-T	6’F-bc^4,3^-T	bc^4,3^-T^b^	6’-diF-bc^4,3^-T	6’F-bc^4,3^-T	bc^4,3^-T^b^

1	d(GGA TGT TCX CGA)	43.5 (−5.2)	46.0 (−2.7)	47.3 (−0.2)	44.5 (−5.5)	46.0 (−4.0)	48.9 (−0.8)
2	d(GGA XGT TCT CGA)	47.1 (−1.6)	47.2 (−1.5)	46.1 (−1.4)	47.9 (−2.1)	47.6 (−2.4)	47.4 (−2.3)
3	d(GGA TGX XCT CGA)	40.3 (−4.2)	41.3 (−3.7)	47.0 (−0.3)	43.3 (−3.4)	42.0 (−4.0)	51.0 (+0.7)
4	d(GCA XXX XXA CCG)	20.2 (−5.4)	30.3 (−3.4)	–	22.0 (−4.5)	22.4 (−4.4)	–

^a^Total strand conc. 2 μM in 10 mM NaH_2_PO_4_, 150 mM NaCl, pH 7.0. *T*_m_ values of unmodified duplexes: DNA1/DNA = 48.7 °C, DNA1/RNA = 50.0 °C, DNA2/DNA = 47.4 °C, DNA2/RNA = 44.4 °C; DNA1 = 5’-d(GGA TGT TCT CGA)-3’, DNA2 = 5’-d(GCA TTT TTA CCG)-3’. ^b^Data taken from ref [36].

An interesting behaviour was observed for the sequence of Table 3, entry 2. There, the ON of the three bc^4,3^-T analogs not only showed almost identical *T*_m_ values when paired to DNA but also to complementary RNA. This means that at least in this sequence context the fluorine atoms had no impact on the duplex stability and the destabilization arose only from the bicyclic scaffold. Whether this finding is a general behaviour or a consequence of the A–X–G nearest neighbour interactions cannot be stated at present.

The base pairing selectivity of the 6’-diF-bc^4,3^-T was evaluated by UV-melting experiment of **ON1** by inserting one of the three possible mismatches (G, C, or T) opposite the modified unit in the otherwise complementary DNA strand (Table 4). As anticipated, the mispairing led to a strong decrease of the melting temperature (*T*_m_ = −8.5 to −13.8 °C) with the GT-Wobble mispair exhibiting the least destabilizing effect. Comparing these values to the ones of the natural system revealed that the modification discriminated the mismatches more efficiently. Consequently, this finding indicated a higher tendency for mismatch discrimination of the 6’-diF-bc^4,3^-T over dT.

**Table 4:** *T*_m_ data [°C] from UV-melting curves (260 nm) of **ON1** in duplex with complementary mismatched DNA.

Entry	Sequence^a^	X = A	X = T	X = G	X = C

**DNA1**	5’-d(GGA TGT TCT CGA)-3’	48.7	39.0	40.2	37.3
**DNA**	5’-d(TCG XGA ACA TCC)-3’		(−9.7)	(−8.5)	(−11.4)
**ON1**	5’-d(GGA TGT TCt CGA)-3’	43.5	29.7	35.0	30.7
**DNA**	5’-d(TCG XGA ACA TCC)-3’		(−13.8)	(−8.5)	(−12.8)

^a^Lowercase letters: modified nucleotide, capital letters: natural DNA. Total strand conc. 2 μM in 10 mM NaH_2_PO_4_, 150 mM NaCl, pH 7.0.

**Figure 4:**
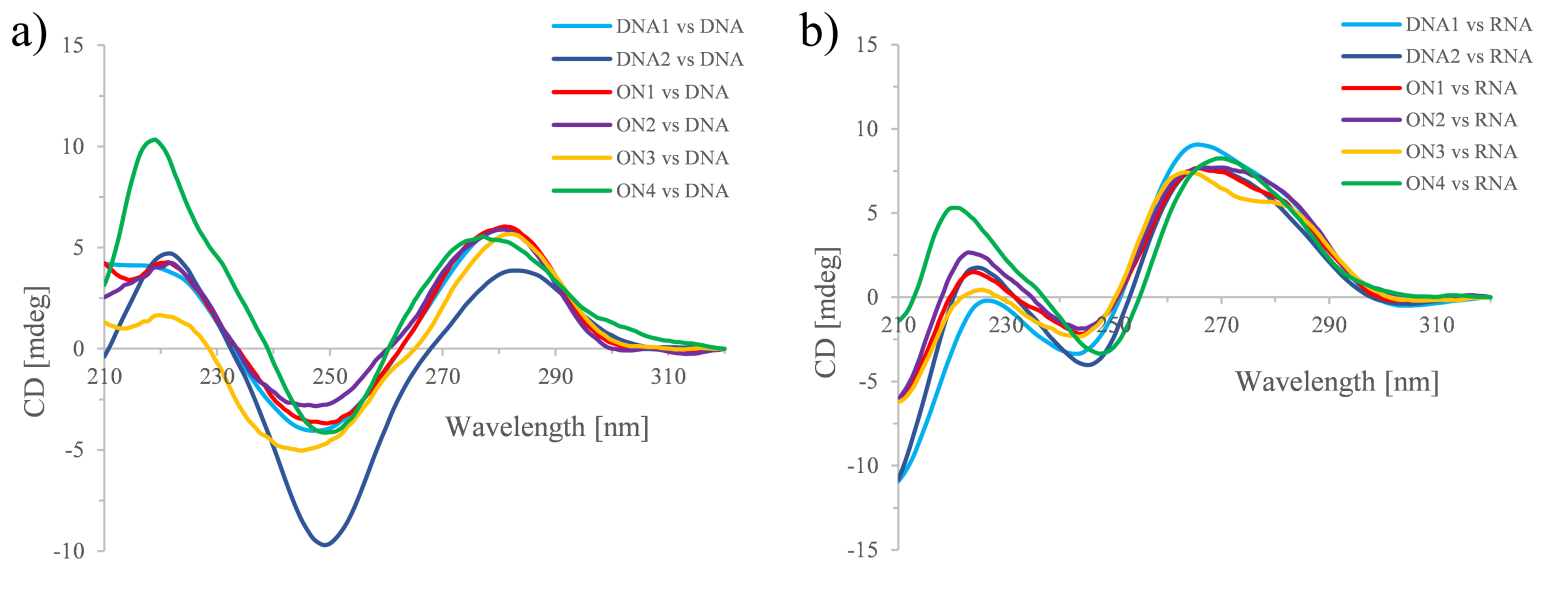
CD spectra of **ON1**–**4** with complementary a) DNA, and b) RNA. Total strand conc. 2 μM in 10 mM NaH_2_PO_4_, 150 mM NaCl, pH 7.0.

To gain more information about the helical structure of **ON1**–**4** hybridized to complementary DNA or RNA, CD spectra of these duplexes were recorded (Figure 4). The duplexes of the four ONs paired to DNA still showed the overall shape of a B-type helix [48], although some duplexes exhibited slight distortions. Modest changes of the ellipticity amplitude maxima in the **ON3**/DNA duplex existed compared to the natural system. Also, some deviation from the natural structure displayed the **ON4**/DNA duplex. There, the band at 245 nm was depressed and the two positive ellipticities (≈220 nm and ≈280 nm) expressed increased intensities. Besides this, the ellipticity at 280 nm was blue-shifted (≈6 nm). All four modified DNA/RNA hybrids exhibited the characteristic shape of an intermediate A/B-type helix [48]. Again, some slight deviations from the natural system could be observed for the 6’-diF-bc^4,3^-T containing duplexes. The most distinct deviations occurred in the case of the **ON4**/RNA duplex. There the band at 225 nm was blue-shifted (≈5 nm) and the one at 245 slight red-shifted (≈2 nm) and both intensities were changed, too. Furthermore, the intensity of the 210 nm peak was reduced.

### RNase H cleavage assay

The most important requirement for an antisense oligonucleotide to induce RNase H activity lies in its DNA-like sugar conformations [49]. This is generally fulfilled by the 6’-diF-bc^4,3^-DNA as well as the 6’F-bc^4,3^-DNA. Furthermore, duplexes of a 6’F-bc^4,3^-modified strand paired to RNA unveiled in the MD simulations a flexible minor groove distance [37]. This flexibility is thought to play a crucial role for the fitting of the duplex into the DNA-binding channel and the phosphate-binding pocket of the enzyme. Furthermore, the phosphate-binding pocket requires a large distortion of the backbone angle α in order that the phosphate group of the AON can be positioned in it [50–51]. The 6’F-bc^4,3^-DNA containing strand also complied with this requirement according to the MD simulations [37]. Therefore, we examined the ability of the 6’F-bc^4,3^-DNA and the 6’-diF-bc^4,3^-DNA to induce the RNase H-mediated cleavage of the complementary RNA strand by utilizing the sequence of **ON4** (5’-d(GCA ttt ttA CCG)-3’) and a chimeric sense strand. The sense strand consisted of five consecutive ribo-A units placed opposite the modified part and 2’-*O*-methyl RNA flanks. The same construct was previously applied for the evaluation of 2’F-tc-ANA [35] and a similar one for CeNA [52]. For the assay *E. coli* RNase H was used due to its commercial availability and its similarity to the human enzyme [22].

**Figure 5:**
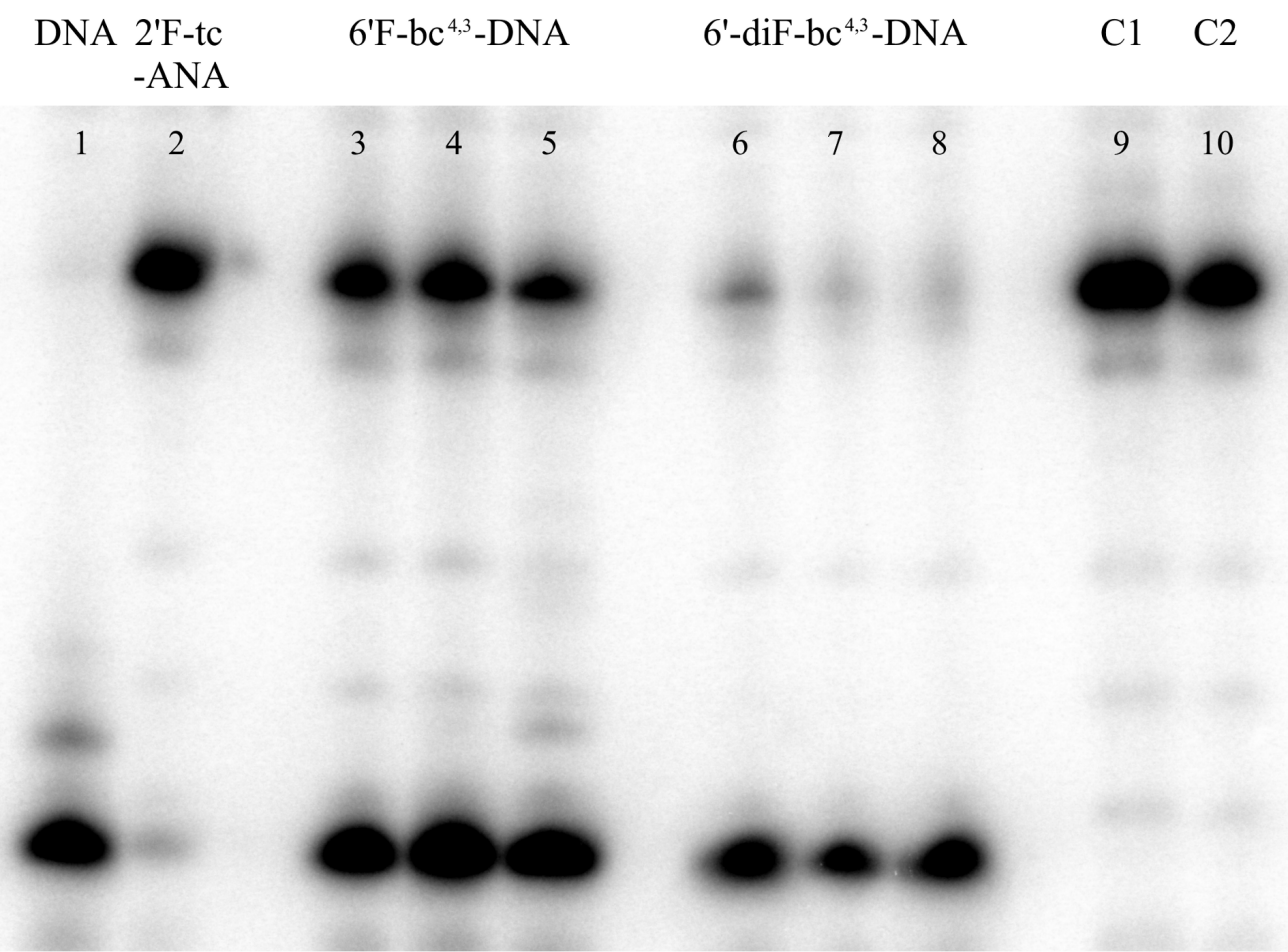
Hydrolysis products of the RNase H activation assay. The DNA served as positive control, whereas C1 (no antisense strand) and C2 (no enzyme) were negative controls.

The cleavage pattern of the RNase H is presented in Figure 5. In the DNA/RNA positive control, the RNA strand was completely cleaved as expected (lane 1). In the negative controls C1 (no antisense strand) and C2 (no enzyme) no degradation of RNA could be observed (lanes 9 and 10). Acceptable substrates for the RNase H were duplexes containing both the 6’F-bc^4,3^-DNA (lanes 3–5) and the 6’-diF-bc^4,3^-DNA (lanes 6–8). The latter was able to induce a more efficient cleavage of the complementary RNA strand than the former. Nevertheless, both modifications recruited the RNase H to a lower extent than natural DNA. The 2’F-tc-ANA modification that has previously been shown to induce RNase H cleavage in a different sequence context was only modestly active (lane 2). Overall, the two new fluorinated bc^4,3^-analogs are among the few modifications which are able to activate RNase H to some extent. These promising results indicate that the 6’-diF-bc^4,3^-DNA and the 6’F-bc^4,3^-DNA may find application in therapeutic gapmer oligonucleotides.

## Conclusion

In this report, we presented the successful synthesis of the 6’-diF-bc^4,3^-T building block where the *gem*-difluorinated bicyclic unit was formed starting from a previously described tricyclic siloxydifluorocyclopropane. The reaction of this tricyclic sugar under NIS-mediated nucleosidation produced two diiodo-substituted intermediates which were reduced by Bu_3_SnH yielding the β-nucleoside **6** as the only diastereoisomer. The crystal structure of nucleoside **6** exhibited the C2’-*endo* conformation of the furanose ring and the *gauche* orientation of the torsions angle γ. Conversion of this nucleoside into the corresponding phosphoramidite building block and its incorporation into oligonucleotides was then successfully achieved. Thermal melting experiment of the modified oligonucleotides paired to complementary DNA or RNA revealed a prominent duplex destabilization for both duplex types (Δ*T*_m_/mod = −1.6 to −5.5 °C). A lesser degree of destabilization was observed for oligonucleotides containing several consecutive modifications hybridized to complementary RNA. The reason for the destabilization might be accounted to repulsive electrostatic interactions between the equatorial fluorine atom and the 5’-oxygen. CD spectroscopy of the duplexes disclosed that the helical structure of the modified oligonucleotides paired to complementary DNA was still of a B-type, whereas an intermediate A/B-type helix was observed for RNA as complement. Furthermore, the RNase H assay of the oligonucleotide containing either five consecutive 6’-diF-bc^4,3^-Ts or 6’F-bc^4,3^-Ts paired to complementary RNA revealed that both modifications were able to recruit this enzyme. In both cases the RNase H cleaved the complementary RNA strand less efficiently as compared to the natural DNA/RNA duplex. This is a promising finding which points to a possible application for both modifications in therapeutic gapmer oligonucleotides.

## Supporting Information

File 1 Additional information, experimental procedures, NMR spectra, and crystallographic data.
