# Supplementary material for: Synthesis, biophysical properties, and RNase H activity of 6’-difluoro[4.3.0]bicyclo-DNA
Source: Beilstein J Org Chem. 2019 Jan 8;15:79–88. doi: 10.3762/bjoc.15.9 (PMC6334804; doi:10.3762/bjoc.15.9)
Supplement: File 1 — Additional information, experimental procedures, NMR spectra, and crystallographic data. [file Beilstein_J_Org_Chem-15-79-s001.pdf]

BEILSTEIN JOURNAL OF ORGANIC CHEMISTRY

Supporting Information

for

Synthesis, biophysical properties, and RNase H activity of 6'-difluoro[4.3.0]bicyclo-DNA

Sibylle Frei, Adam K. Katolik and Christian J. Leumann

Beilstein J. Org. Chem. **2019,** *15,* 79–88. doi:10.3762/bjoc.15.9

Additional information, experimental procedures, NMR spectra, and crystallographic data

License and Terms: This is a supporting information file under the terms of the Creative Commons Attribution License (http://creativecommons.org/licenses/by/4.0). Please note that the reuse, redistribution and reproduction in particular requires that the authors and source are credited.
The license is subject to the *Beilstein Journal of Organic Chemistry* terms and conditions: (https://www.beilstein-journals.org/bjoc)

Table of Contents

1. Formation of the *gem*-difluorinated [4.3.0]-bicyclo sugar……………………….. S3

2. Structural parameters of the 6'-diF-bc^{4,3} nucleosides…………………………… S3

3. Experimental part…………………………………………………………………... S6

3.1 General…………………………………………………………………………... S6

3.2 UV-melting curves……………………………………………………………… S6

3.3 Circular dichroism spectroscopy……………………………………………… S7

3.4 DFT calculations………………………………………………………………… S7

3.5 Oligonucleotide synthesis and purification……………………………………… S7

3.6 RNase H degradation experiment……………………………………………….. S10

3.7 Numbering of the sugars and nucleosides……………………………………….S11

3.8 Synthesis and characterization………………………………………………….... S12

4. NMR spectra of the new compounds……………………………………………... S20

5. X-ray crystal structure analysis…………………………………………………… S34

6. References…………………………………………………………………………... S46

1. Formation of the *gem*-difluorinated [4.3.0]bicyclo sugar

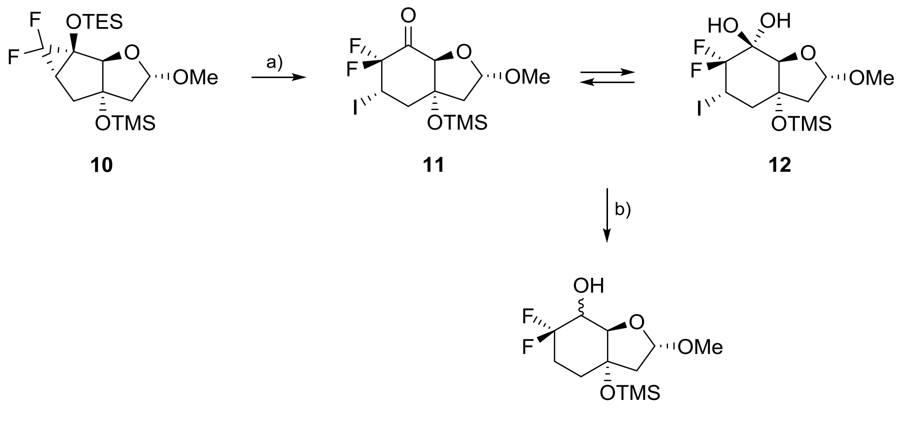

Scheme S1: Synthesis of the *gem*-difluorinated bicyclic sugar **13**. Conditions: a) NIS, DCM, 0 °C to rt, 2.6 h, 60%; b) Bu_3SnH, AIBN, toluene, 90 °C, 30 min, 62%.

2. Structural parameters of the 6'-diF-bc^{4,3} nucleosides

Table S1: Distance of the fluorine atoms to the 5'-oxygen or 5'-hydrogen atoms.

	F···O-C(5') [Å]		F···HO-C(5') [Å]		F···H-C(5') [Å]	
Fluorine Atom / Nucleoside	F_a	F_b	F_a	F_b	F_a	F_b
**6a**[a]	2.80	3.59	2.50	3.78	2.54	2.54
**6b**[a]	2.73	3.57	2.61	3.84	2.56	2.51
**7**[b]	2.61	3.55	2.20	3.61	2.77	2.34

[a]Data of the crystal structure. [b]Data of the calculated (MP2, 6-311G*) minimal energy conformer.

Table S2: C–C bond lengths in the carbocyclic ring of the 6'-diF-bc^{4,3}-T and the bc^{4,3}-T.

Nucleoside	C3'-C4' [Å]	C4'-C5' [Å]	C5'-C6' [Å]	C6'-C7' [Å]	C7'-C8' [Å]	C8'-C3' [Å]
**6a**[a]	1.55	1.53	1.50	1.49	1.53	1.53
**6b**[a]	1.54	1.53	1.51	1.49	1.53	1.52
**7**[b]	1.54	1.52	1.54	1.52	1.53	1.53
bc^{4,3}-T (a)[a,c]	1.52	1.53	1.52	1.52	1.46	1.52
bc^{4,3}-T (b)[a,c]	1.54	1.53	1.52	1.52	1.50	1.53

[a]Data of the crystal structure. [b]Data of the calculated (MP2, 6-311G*) minimal energy conformer. [c]The structures a and b were two different molecules in the same unit. Data taken from ref [1].

Table S3: Bond angles in the carbocyclic ring of the 6'-diF-bc^{4,3}-T and the bc^{4,3}-T.

Nucleoside	C3'-C4'-C5' [°]	C4'-C5'-C6' [°]	C5'-C6'-C7' [°]	C6'-C7'-C8' [°]	C7'-C8'-C3' [°]	C8'-C3'-C4' [°]	X-C6'-X[a] [°]	H-C7'-H [°]	H-C8'-H [°]
**6a**[b]	115	109	114	110	115	114	105	108	108
**6b**[b]	116	110	114	110	114	114	105	108	108
**7**[c]	112	108	115	111	109	113	106	108	108
bc^{4,3}-T (a)[a,d]	115	109	111	113	116	115	108	108	107
bc^{4,3}-T (b)[a,d]	116	108	110	111	116	115	108	108	108

[a]**6a/b**, **7**: X = F, bc^{4,3}-T: X = H. [b]Data of the crystal structure. [c]Data of the calculated (MP2, 6-311G*) minimal energy conformer. [d]The structures a and b were two different molecules in the same unit. Data taken from ref [1].

3. Experimental part

3.1 General

The chemicals used for the synthesis of the phosphoramidite building block were purchased from Sigma-Aldrich, Acros Organics, TCI or ABCR. All reactions were performed in oven-dried glassware under an atmosphere of argon. The anhydrous solvents for the reactions were obtained by filtration through activated alumina or by storage over activated molecular sieves (4 Å). Column chromatography (CC) was carried out on silica gel (Sigma-Aldrich, pore size 60 Å, 230–400 mesh, neutralized with 0.1% w/Ca) as stationary phase. The solvents used for CC were of technical grade and distilled prior to use. Thin-layer chromatography (TLC) was conducted on silica gel plates (Marchery-Nagel, SIL G-25 UV_{254}). The visualisation of the compounds was achieved either under UV light or by staining in dip solution [*p*-anisaldehyde (10 ml), acetic acid (2 ml), concentrated H_2SO_4 (10 ml), and ethanol (180 ml)], followed by heating with a heat gun. The NMR spectra were recorded on a Bruker Avance II 400, Bruker Avance III HD 300 or Bruker Avance III HD 400 spectrometer at 400 MHz (^{1}H), at 101 MHz (^{13}C), at 376 MHz (^{19}F) and at 162 MHz (^{31}P) in either $CDCl_3$ or $(CD_3)_2SO$. The chemical shifts (δ) are reported relative to the undeuterated residual solvent peak [$CHCl_3$: 7.26 ppm (^{1}H) and 77.16 ppm (^{13}C); CHD_2CD_3SO: 2.50 ppm (^{1}H) and 39.52 ppm (^{13}C)]. The signal assignments are based on APT,(^{1}H,^{1}H) and (^{1}H,^{13}C) correlation experiments. The chemical shifts of ^{19}F and ^{31}P NMR are reported relative to 80% $CFCl_3$ or 85% H_3PO_4 as the external standard. High-resolution mass detections were performed by electrospray ionization on a LTQ Orbitrap XL spectrometer in the positive mode (ion trap, ESI^+).

3.2 UV-melting curves

The UV-melting curves were measured on a Varian Cary Bio 100 UV–vis spectrometer. All experiments were performed with a duplex concentration of 2 μM in a NaCl (150 mM)/NaH_2PO_4 (10 mM) buffer (pH 7). To avoid the evaporation of the solvent during the measurement the samples were covered with a layer of dimethylpolysiloxane. The absorbance was monitored at 260 nm. For every sample three cooling-heating circles (80 → 15 or 0 °C) were performed with a temperature gradient of 0.5 °C/min. T_m values were obtained from the maxima of the curves first derivative and were extracted with the Varian WinUV software. They were reported as an average of at least five ramps.

3.3 Circular dichroism spectroscopy

The CD spectra were measured on a Jasco J-715 spectropolariometer equipped with a Jasco PFO-350S temperature controller using the Spectra Manager v1.54 software. The same sample conditions were used as for the UV-melting curves. All spectra were recorded in the range of 210–320 nm at a scanning speed of 50 nm/min at 20 °C. They were baseline-corrected with a blank spectra (NaCl (150 mM)/NaH_2PO_4 (10 mM) buffer, pH 7), smoothed and normalized (320 nm = 0 mdeg). The reported spectra correspond to the average of three scans.

3.4 DFT calculations

The conformational analysis of nucleoside **7** was carried out with the Gaussian 09 software [2] by using the second order Møller-Plesset perturbation (MP2) theory and the 6-311G* basis set. The potential energy profile was achieved in varying the pseudorotation phase angle *P* from 0–360° (in 10° steps) at a certain range of the maximum puckering amplitude ν_{max} (25°–50° in 5° steps). The output was visualised with the OriginPro 2016 software [3].

3.5 Oligonucleotide synthesis and purification

The oligonucleotide synthesises were carried out on a LBK Gene Assembler Plus (Pharmacia) DNA synthesiser in a 1.3 μM scale. The solid support (dA-Q-CPG 500, dmf-dG-Q-CPG 500) and the natural DNA phosphoramidites (dT, dC^{4bz}, dG^{2DMF}, dA^{6Bz}) were purchased from Glen Research. The natural DNA phosphoramidites were prepared as a 0.1 M solutions in ACN and were coupled by using a 90 s step. The 6'-diF-$bc^{4,3}$-DNA phosphoramidite was prepared as 0.15 M solution in ACN and the coupling time was extended to 12 min. As a coupling agent 5-(ethylthio)-1*H*-tetrazole (0.25 M in ACN) was used. The capping was performed with a solution of DMAP in ACN (0.5 M, Cap A) and a solution of 25% Ac_2O and 12.5% *sym*-collidine in ACN (Cap B). For the oxidation step a solution of 1.1 M TBHP in DCM was used. The detritylation step was conducted with a solution of 3% dichloroacetic acid in dichloroethane.

The cleavage from the solid support and the deprotection of the oligonucleotides was achieved by using standard conditions (concentrated ammonia at 55 °C for 16 h). Afterwards the tubes were centrifuged, the supernatants were collected, and the remaining beads were washed with MQ-water (3 × 500 μl). The combined supernatants were filtered using spin-

columns (Amicon Ultra 0.5 ml centrifugal filters, MWCO 3kDa) and concentrated to dryness a the Speed-Vac (UniVapo 150, UniEquip).

The crude oligonucleotides were purified by ion-exchange HPLC (DNAPac PA200, Dionex) on an ÄktaTM basic 10/100 system (Amersham Pharmacia Biotech). As mobile phase the following two buffers were used: (A) 25 mM Trizma in H_2O, pH 8, and (B) 25 mM Trizma, 1.25 M NaCl in H_2O, pH 8. A linear gradient of B in A was applied over 50 min with a flow rate of 1 ml/min and detection at 260 nm. The purified oligonucleotides were desalted using Sep-Pak Classic C18 Cartridges (Waters), quantified with a Nanodrop spectrophotometer (260 nm) and analyzed by LC–MS. The purities were measured by HPLC. The natural DNA and RNA oligonucleotides were purchased from Microsynth AG and re-purified by HPLC and desalted if necessary.

Table S4: List of the synthesized oligonucleotides containing the 6'-diF-bc[4,3]-T and their characterization by LC-MS and ion exchange HPLC.

Entry	Sequence[a]	m/z_{calcd}	m/z_{found}	HPLC Gradient[b]	Retention Time[c] [min]	Purity [%]
**ON1**	5'-d(GGA TGT TCt CGA)-3'	3752.5	3751.8	B: 30 → 45%	11.90	97.6
**ON2**	5'-d(GGA tGT TCT CGA)-3'	3752.5	3751.0	B: 25 → 40%	13.07	95.2
**ON3**	5'-d(GGA TGt tCT CGA)-3'	3828.5	3827.0	B: 32 → 45%	12.99	100.0
**ON4**	5'-d(GCA ttt ttA CCG)-3'	3991.6	3990.1	B: 30 → 40%	13.93	100.0

[a]Capital letters: natural nucleotides, lowercase letters: modified nucleotides. [b]Mobile phase A: 25 mM Trizma in H_2O, pH 8; mobile phase B: 25 mM Trizma, 1.25 M NaCl in H_2O, pH 8. [c]For all ONs: B = 30 → 60%, length CV = 4; flow rate 1 ml/min.

3.6 RNase H degradation experiment

Preparation of stock solutions

Both the positive control antisense strand and the chimeric sense strand were ordered from Microsynth AG. To provide better presentability of results, the sense strand was additionally re-purified using 20% polyacrylamide gel electrophoresis (acrylamide/bis acrylamide 29:1). The gel bands were eluted three times with 3 ml dH_2O (once shaking at ambient temperature overnight and twice vortexing briefly), and the eluted oligomer was desalted with a Sep-Pac C18 Classic Cartridge (360 mg Sorbent per Cartridge, 55–105 μm Particle Size, 50/pk), and concentrated. It was then re-dissolved with dH_2O, quantified with a Nanodrop ND-1000 Spectrophotometer (Witec AG), and aliquoted to form a usable 1 μM stock solution. Regarding the antisense compounds, 10 μM stock solutions were prepared in the same manner.

Radiolabeling of the sense strand

A 20 μl reaction was prepared featuring: (i) 100 pmol of the sense strand, (ii) gamma ^{32}P ATP, 1X polynucleotide kinase buffer (50 mM Tris-HCl, 10 mM $MgCl_2$, 5 mM DTT, 0.1 mM spermidine, pH 7.6 at 25 °C), and (iii) T4 polynucleotide kinase (EK0031, Thermo Scientific, added last). This reaction was shaken for 1 h at 37 °C, and subsequently the resulting radiolabeled oligomer was filtered using an Amicon Ultra- 0.5 ml centrifugal filters to remove unreacted gamma ^{32}P ATP.

RNase H reactions

All reactions proceeded in a 20 μl total volume and featured: (i) an adequate quantity (> 100 K cpm) of the 5´ radiolabeled sense strand, (ii) a not radiolabeled ("cold") sense strand (0.1 μM), (iii) an antisense strand (2.5 μM), (iv) 1x RNase H reaction buffer (20 mM Tris-HCl, 40 mM KCl, 8 mM $MgCl_2$, 1 mM DTT, pH 7.8). This mixture was heated to 95 °C for 5 min, and then gradually cooled to rt and subsequently 4 °C over several hours to ensure annealing. Next, the *E. coli* RNase H (0.2 Units/μl, EN0201, Thermo Scientific) was added and the reactions were incubated overnight (approximately 15 h) at 22 °C with mechanical shaking. Next, an equal volume (20 μl) of formamide gel loading buffer was added to denature the strands, and the vials were heated to 95 °C for 5 min. 5 μl of this mixture was then added to a lane of a previously prepared 32 cm × 42 cm × 0.2 mm polyacrylamide gel (20%, 29:1 acrylamide/bis acrylamide, 1 × TBE, and 7 M Urea). The gel was then inserted into a gel apparatus (Model S2 BRL) with 0.5X TBE buffer in both upper and lower chambers, and connected to a power supply (Life Technologies GIBCO BRL PS 9009) under a constant power (40 W) and variable voltage regime for approximately 3 h. Next, the gel was opened

and placed inside a cassette along with a phosphorimaging plate (Fujifilm), and frozen at -20 °C overnight. Finally, the exposed plate was inserted into a Typhoon Phosphorimager and scanned.

3.7 Numbering of the sugars and nucleosides

Sugars

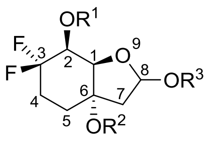

Nucleosides

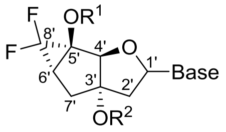

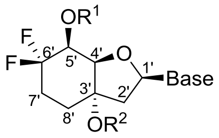

3.8 Synthesis and characterization

1-[(2'*R* and *S*,3'*R*,5'*R*,6'*S*)-2'-Deoxy-3',5'-ethano-5'-*O*-triethylsilyl-8',8'-difluoro-2'-iodo-5',6'-methano-3'-*O*-trimethylsilyl-α and β-D-ribofuranosyl]thymine (2α/β)

and

1-[(2'*R*,3'*R*,7'*R*)-2'-deoxy-6'6'-difluoro-2',7'-diiodo-3'-*O*-trimethylsilyl-3',5'-propano-5'-oxo-β-D-ribofuranosyl]thymine (3)

and

1-[(2'*R*,3'*R*,7'*R*)-2'-deoxy-6',6'-difluoro-5',5'-dihydroxy-2',7'-diiodo-3'-*O*-trimethylsilyl-3',5'-propano-β-D-ribofuranosyl]thymine (4)

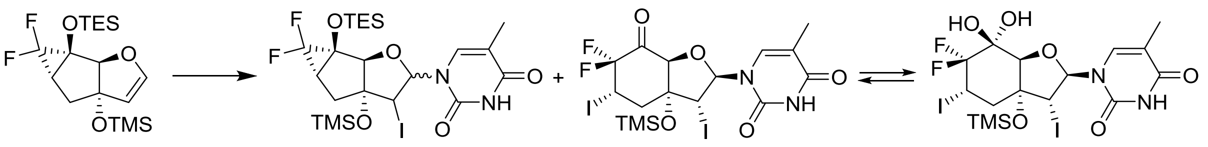

1 **2α/β** **3** **4**

BSA (0.46 ml, 1.88 mmol, 4.5 equiv) was added dropwise to a suspension of thymine (159 mg, 1.26 mmol, 3 equiv) in dry DCM (1.5 ml) at rt. The mixture was stirred for 1 h before a solution of the glycal **1** (159 mg, 0.42 mmol) in dry DCM (0.8 ml) was slowly added. The resulting mixture was cooled down to 0 °C and NIS (189 mg, 0.84 mmol, 2.0 equiv) was added in three portions over a period of 35 min. Afterwards the reaction mixture was stirred for an additional 30 min at 0 °C and for 25.5 h at rt. The red solution was then diluted with Et_2O (10 ml) and washed with sat. $Na_2O_3S_2$ sol. (2 × 10 ml) and sat. $NaHCO_3$ sol. (10 ml). The combined aqueous phase was extracted with Et_2O (3 × 30 ml). The combined organic phase was dried ($MgSO_4$), concentrated and dried at HV. The crude product (385 mg, >100%) was subjected to the next reaction without further purification.

Data for **2α/β**: R_f = 0.38 (hex/Et_2O 1:1); ^{1}H NMR (400 MHz, $CDCl_3$) δ 8.93 (*s*, 1H, H-N(3)), 8.89 (*s*, 1H, H-N(3)), 7.48 (*d*, *J* = 1.4 Hz, 1H, H-C(6)), 7.20 (*d*, *J* = 1.4 Hz, 1H, H-C(6)), 6.51 (*d*, *J* = 10.0 Hz, 1H, H-C(1')), 6.36 (*d*, *J* = 6.5 Hz, 1H, H-C(1')), 4.55 (*s*, 1H, H-C(4')), 4.40 (*d*, *J* = 6.5 Hz, 1H, H-C(2')), 4.33 (*s*, 1H, H-C(4')), 4.13 (*d*, *J* = 10.0 Hz, 1H, H-C(2')), 2.83 – 2.67 (*m*, 1H, H-C(7')), 2.21 – 2.00 (*m*, 4H, 2x H-C(6'), 2x H-C(7')), 1.96 (*d*, *J* = 1.4 Hz, 6H, 2x *Me*-C(5)), 1.70 – 1.59 (*m*, 1H, H-C(7')), 1.00 – 0.87 (*m*, 18H, 2x ($CH_3CH_2)_3$Si), 0.74 – 0.56 (*m*, 12H, 2x ($CH_3CH_2)_3$Si), 0.21 (*s*, 9H, *Me*$_3$Si), 0.17 (*s*, 9H, *Me*$_3$Si); ^{13}C NMR (101 MHz, $CDCl_3$) δ 163.6, 163.6 (C(4)), 150.7, 150.0 (C(2)), 135.2, 135.0 (C(6)), 113.8 (*dd*, *J* = 306.6, 290.5 Hz, C(8')), 113.5 (*dd*, *J* = 310.5, 287.1 Hz, C(8')), 112.0, 111.9 (C(5)), 93.6 – 93.2 (*m*, C (1'),

C(3’)), 93.0 (*d*, *J* = 4.7 Hz, C(3’)), 90.1 (C(1’)), 88.2 (C(4’)), 84.5 (*d*, *J* = 3.7 Hz, C(4’)), 67.0 – 66.7 (*m*, C(5’)), 66.8 (*dd*, *J* = 11.1, 8.1 Hz, C(5’)), 36.2 (C(2’)), 36.1 (*d*, *J* = 2.6 Hz, C(7’)), 35.6 (C(2’)), 34.5 (*dd*, *J* = 11.5, 8.6 Hz, C(6’)), 32.6 (*dd*, *J* = 12.0, 9.5 Hz, C(6’)), 28.3 (*d*, *J* = 4.9 Hz, C(7’)), 13.0, 12.8 (*Me*-C(5)), 6.7, 6.6 ((CH$_3$CH$_2$)$_3$Si), 5.2 ((CH$_3$$CH_2$)$_3$Si), 4.9 (*d*, *J* = 2.0 Hz, (CH$_3$$CH_2$)$_3$Si), 2.1, 1.8 (*Me*$_3$Si); ^{19}F NMR (376 MHz, CDCl$_3$) δ -134.8 (*dd*, *J* = 163.3, 16.4 Hz, 1F), -135.1 (*dd*, *J* = 163.2, 16.6 Hz, 1F) -144.9 (*d*, *J* = 163.2 Hz, 1F), -145.9 (*d*, *J* = 163.3 Hz, 1F); ESI$^+$-HRMS *m/z* calcd for $C_{22}H_{36}O_5N_2F_2ISi_2$ [(M + H)$^+$] 629.1170, found 629.1173.

Data for **3**/**4**: R$_f$ = 0.27 (hex/Et$_2$O 1:5); ^{1}H NMR (400 MHz, CDCl$_3$) δ 9.40 (*s*, 1H, N*H*), 8.80 (*s*, 1H, N*H*), 8.41 (*br*, 1H, O*H*), 7.50 (*d*, *J* = 3.5 Hz, 1H, O*H*), 7.36 (*d*, *J* = 1.3 Hz, 1H, H-C(6)), 7.05 (*d*, *J* = 1.3 Hz, 1H, H-C(6)), 6.43 (*d*, *J* = 9.4 Hz, 1H, H-C(1’)), 5.49 (*d*, *J* = 9.8 Hz, 1H, H-C(1’)), 5.16 (*d*, *J* = 9.8 Hz, 1H, H-C(2’)), 5.02 (*d*, *J* = 5.6 Hz, 1H, H-C(4’)), 4.57 (*ddd*, *J* = 26.1, 13.5, 5.4 Hz, 1H, H-C(7’)), 4.35 (*d*, *J* = 2.9 Hz, 1H, H-C(4’)), 4.08 – 3.94 (*m*, 1H, H-C(7’)), 3.96 (*d*, *J* = 9.4 Hz, 1H, H-C(2’)), 2.88 – 2.67 (*m*, 3H, H-C(8’)), 2.47 (*t*, *J* = 14.0 Hz, 1H, H-C(8’)), 1.97 (*d*, *J* = 1.3 Hz, 3H, *Me*-C(5)), 1.95 (*d*, *J* = 1.3 Hz, 3H, *Me*-C(5)), 0.32 (*s*, 9H, *Me$_3$*Si), 0.26 (s, 9H, *Me$_3$*Si); ^{13}C NMR (101 MHz, CDCl$_3$) δ 190.2 (*dd*, *J* = 30.0, 23.4 Hz, C(5’)), 164.2, 164.1 (C(4)), 151.4, 150.8 (C(2)), 139.8, 135.8 (C(6)), 118.0 (*dd*, *J* = 258.7, 247.6 Hz, C(6’)), 114.2 (*dd*, J = 265.0, 248.6 Hz, C(6’)), 112.5, 112.1 (C(5)), 96.0, 91.4 (C(1’)), 90.9 (*dd*, *J* = 28.7, 21.4 Hz, C(5’)), 85.7 (*d*, *J* = 3.4 Hz, C(4’)), 85.1 – 85.0 (*m*, C(4’)), 84.4, 80.7 (C(3’)), 41.6 (*d*, *J* = 4.4 Hz, C(8’)), 40.96 (*d*, *J* = 3.6 Hz, C(8’)), 31.7, 29.9 (C(2’)), 17.4 (*t*, *J* = 23.9 Hz, C(7’)), 15.6 – 14.7 (*m*, C(7’)), 12.7, 12.4 (*Me*-C(5)), 1.8, 1.7 (*Me$_3$*Si); ^{19}F NMR (376 MHz, CDCl$_3$) δ -107.9 (*dd*, *J* = 242.5, 3.4 Hz, 1F), -114.1 (*d*, J = 244.6 Hz, 1F), -114.4 (*ddd*, *J* = 242.5, 27.9, 4.7 Hz), -121.41 (*dd*, *J* = 244.6, 26.0 Hz, 1F); ESI$^+$-HRMS *m/z* calcd for $C_{16}H_{21}O_5N_2F_2I_2Si$ [(M + H)$^+$] 640.9272, found 640.9250; ESI$^+$-HRMS *m/z* calcd for $C_{16}H_{23}O_6N_2F_2I_2Si$ [(M + H)$^+$] 658.9377, found 658.9354.

1-[(3'*S*,5'*R*,6'*S*)-2'-Deoxy-3',5'-ethano-5'-*O*-triethylsilyl-8',8'-difluoro-5',6'-methano-3'-O-trimethylsilyl-α,β-D-ribofuranosyl]thymine (5α/β)

and

1-[(3'*S*,5'*S*)-2'-deoxy-6',6'-difluoro-3'-*O*-trimetylsilyl-3',5'-propano-β-D-ribofuranosyl]thymine (6)

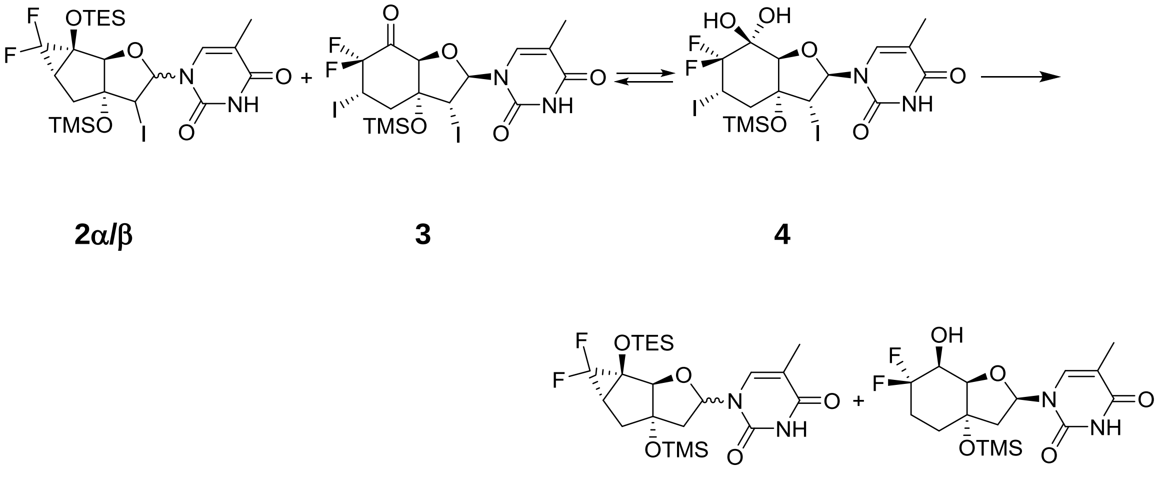

2α/β **3** **4**

5α/β **6**

To a suspension of the crude product (385 mg, 0.42 mmol) in dry toluene (4.2 ml) was added Bu_3SnH (0.40 ml, 1.49 mmol, 3.5 equiv) and AIBN (7 mg, 0.04 mmol, 0.1 equiv) at rt. Then the reaction mixture was heated up to 90 °C and was stirred for 30 min at this temperature. After cooling down to rt the solvent was removed under reduced pressure. The residue was adsorbed on silica gel and purified by CC (hex/Et_2O 2:1 → 1:5) to yield an anomeric mixture of the tricyclic nucleoside **5α/β** (71 mg, 0.14 mmol, 34% over two steps, α/β-ratio 1:4.5) and the bicyclo nucleoside **6** (79 mg, 0.20 mmol, 48% over two steps). The crystals of **6** which were suitable for X-ray diffraction analysis were obtained by recrystallization in hexane.

Data for **5α/β**: they were in accordance with the literature [4].

Data for **6**: R_f = 0.40 (hex/Et_2O 1:10); ^{1}H NMR (400 MHz, DMSO-d_6) δ 11.30 (*s*, 1H, H-N(3)), 8.05 (*d*, *J* = 1.1 Hz, 1H, H-C(6)), 6.47 (*d*, *J* = 6.3 Hz, O*H*), 6.15 (*dd*, *J* = 8.6, 5.6 Hz, 1H, H-C(1')), 4.14 – 3.79 (*m*, 2H, H-C(4'), H-C(5')), 2.28 – 2.06 (*m*, 2H, H-C(7'), H-C(8')), 2.24 (*dd*, *J* = 12.7, 8.6 Hz, 1H, H-C(2')), 2.10 (*dd*, *J* = 12.7, 5.6 Hz, 1H, H-C(2')), 1.93 – 1.81 (*m*, 2H, H-C(7'), H-C(8')), 1.77 (*d*, *J* = 1.1 Hz, 3H, *Me*-C(5)), 0.16 (s, 9H, Me_3Si); ^{13}C NMR (101 MHz, DMSO-d_6) δ 163.7 (C(4)), 150.6 (C(2)), 136.6 (C(6)), 123.3 (*dd*, *J* = 250.1, 240.9 Hz, C(6')), 108.4 (C(5)), 84.3 (C(1')), 83.9 (*d*, *J* = 5.6 Hz, C(4')), 79.7 (C(3')), 67.5 (*dd*, *J* = 29.5, 21.3 Hz, C(5')), 41.8 (C(2')), 29.7 – 29.4 (*m*, C(8')), 25.3 (*t*, *J* = 23.3 Hz, C(7')), 12.3 (*Me*-C(5)), 2.0

(Me_3Si); ^{19}F NMR (376 MHz, DMSO-d_6) δ -104.6 (*d*, *J* = 245.2 Hz, 1F), -108.9 (*dd*, *J* = 244.7, 22.4 Hz, 1F); ESI$^+$-HRMS *m/z* calcd for $C_{16}H_{25}O_5N_2F_2Si$ [(M + H)$^+$] 391.1495, found 391.1493.

1-[(3'*S*,5'*S*)-2'-Deoxy-6',6'-difluoro-3',5'-propano-β-D-ribofuranosyl]thymine (7)

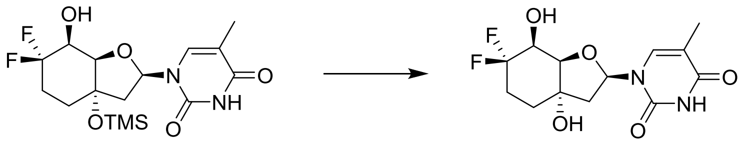

6 **7**

The nucleoside **6** (1.119 g, 2.87 mmol) was dissolved in a mixture of dry DCM (27 ml) and dry pyridine (5.4 ml). The solution was then cooled to 0 °C. HF-pyridine (≈70% HF, 1.5 ml, 57.72 mmol, 20.1 equiv) was added dropwise, and the reaction mixture was stirred at 0 °C for 2.3 h. Afterwards it was adsorbed on silica gel (6.7 g), concentrated and purified by CC (hex/EtOAc 1:1 → 1:40). The free nucleoside **7** (583 mg, 1.83 mmol, 64%) was isolated as white foam.

Data for **7**: R_f = 0.23 (10% MeOH in DCM); ^{1}H NMR (400 MHz, DMSO-d_6) δ 11.26 (*s*, 1H, N*H*), 8.11 (*d*, *J* = 1.2 Hz, 1H, H-C(6)), 6.45 (*d*, *J* = 6.0 Hz, 1H, *H*O-C(5')), 6.22 (*dd*, *J* = 9.1, 5.5 Hz, 1H, C(1')), 5.33 (*s*, 1H, *H*O-C(3')), 3.93 (*dq*, *J* = 10.3, 5.2 Hz, 1H, H-C(5')), 3.86 (*t*, *J* = 4.3 Hz, 1H, H-C(4')), 2.27 – 2.09 (*m*, 1H, H-C(7')), 2.14 (*dd*, *J* = 12.4, 9.1 Hz, 1H, H-C(2')), 2.06 – 1.98 (*m*, 1H, H-C(8')), 1.96 (*dd*, *J* = 12.4, 5.5 Hz, 1H, H-C(2')), 1.91 – 1.80 (*m*, 1H, H-C(7')), 1.76 (*d*, *J* = 1.2 Hz, 3H, *Me*-C(5)), 1.80 – 1.70 (*m*, 1H, H-C(8')); ^{13}C NMR (101 MHz, DMSO-d_6) δ 163.8 (C(4)), 150.7 (C(2)), 136.9 (C(6)), 123.7 (*dd*, *J* = 250.7, 241.0 Hz, C(6')), 108.6 (C(5)), 84.3 (C(1')), 83.5 (*d*, *J* = 6.4 Hz, C(4')), 75.9 (C(3')), 67.9 (*dd*, *J* = 30.2, 21.4 Hz, C(5')), 41.6 (C(2')), 29.6 (*dd*, *J* = 7.9, 3.6 Hz, C(8')), 25.4 (*t*, *J* = 23.0 Hz C(7')), 12.3 (*Me*-C(5)); ^{19}F NMR (376 MHz, DMSO-d_6) δ -103.0 (*d*, *J* = 244.5 Hz, 1F), -108.3 (*dd*, *J* = 244.5, 28.7 Hz, 1F); ESI$^+$-HRMS *m/z* calcd for $C_{13}H_{17}O_5N_2F_2$ [(M + H)$^+$] 319.1100, found 319.1098.

1-{(3'*S*,5'*S*)-2'-Deoxy-6',6'-difluoro-5'-*O*-[(4,4'-dimethyoxytriphenyl)methyl]-3',5'-propano-β-D-ribofuranosyl}thymine (8)

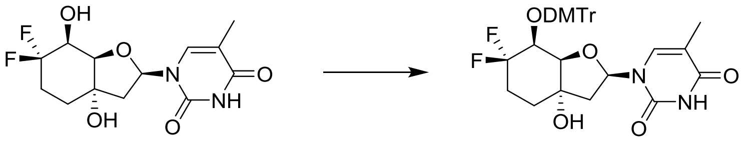

7 **8**

A solution of DMTr-Cl (6.1, 18 mmol) in dry DCM (15 ml) was added to a suspension of AgOTf (4.6 g, 17.9 mmol) in dry DCM (3 ml). The resulting mixture was stirred for 3.5 h at rt. Four equivalent portion of the supernatant (3.16 ml, 3.14 mmol, 2 equiv) were added to a solution of the free nucleoside **7** (502 mg, 1.58 mg) in a mixture of dry DCM (2.8 ml) and dry pyridine (5.6 ml) over a period of 3.25 h. The reaction mixture was then stirred for 22.2 h at rt. Afterwards it was diluted with EtOAc (100 ml) and washed with sat. $NaHCO_3$ sol. (2 × 100 ml). The combined aqueous phase was extracted with EtOAc (5 × 200 ml). The combined organic phases were dried ($MgSO_4$) and concentrated. Purification by CC (1% MeOH in DCM + 0.5% Et_3N) afforded the DMTr-protected nucleoside **8** (549 mg, 0.88 mmol, 56%) as a white foam.

Data for **8**: R_f = 0.29 (5% MeOH in DCM); ^{1}H NMR (400 MHz, DMSO-d_6) δ 11.43 (*s*, 1H, N*H*), 7.55 – 7.47 (*m*, 3H, H-C(6), H-arom), 7.40 – 7.35 (*m*, 2H, H-arom), 7.35 – 7.28 (*m*, 4H, H-arom), 7.27 – 7.21 (*m*, 1H, H-arom), 6.92 – 6.88 (*m*, 4H, H-arom), 5.77 (*dd*, *J* = 8.3, 4.8 Hz, 1H, H-C(1')), 5.25 (*s*, 1H, O*H*), 3.75 (*s*, 6H, 2x *Me*O), 3.75 – 3.65 (*m*, 1H, H-C(5')), 3.40 (*t*, *J* = 4.1 Hz, 1H, H-C(4')), 2.16 (*dd*, *J* = 13.7, 8.3 Hz, 1H, H-C(2')), 1.98 (*dd*, *J* = 13.7, 4.8 Hz, 1H, H-C(2')), 1.93 – 1.82 (*m*, 1H, H-C(7')), 1.84 (*d*, *J* = 1.2 Hz, 3H, *Me*-C(5)), 1.75 – 1.58 (*m*, 3H, H-C(7'), 2x H-C(8')); ^{13}C NMR (101 MHz, DMSO-d_6) δ 163.6 (C(4)), 158.3, 158.3 (C-arom), 150.2 (C(2)), 145.5 (C-arom), 135.8, 135.4 (C-arom), 134.9 (*d*, *J* = 3.3 Hz, C(6)), 130.4, 130.2, 127.9, 127.6, 126.9 (*C*H-arom), 121.4 (*dd*, *J* = 247.6, 246.0 Hz, C(6')), 113.0 (*C*H-arom), 109.2 (C(5)), 87.5 (*C*(Ph)$_3$), 82.6 (C(1')), 82.0 (*d*, *J* = 8.5 Hz, C(4')), 74.6 (C(3')), 69.49 – 68.38 (*m*, C(5')), 55.0 (*Me*O-DMTr), 44.5 (C(2')), 30.9 (*d*, *J* = 8.6 Hz, C(8')), 29.4 (*t*, *J* = 23.3 Hz, C(7')), 12.5 (*Me*-C(5)); ^{19}F NMR (376 MHz, DMSO-d_6) δ -99.9 (*d*, *J* = 238.7 Hz, 1F), -110.9 – -112.0 (*m*, 1F); ESI$^+$-HRMS *m/z* calcd for $C_{34}H_{34}O_7N_2F_2Na$ [(M + Na)$^+$] 643.2226, found 643.2214.

1-{(3'*S*,5'*S*)-3'-*O*-[(2-Cyanoethoxy)diisopropylaminophosphanyl]-2'-deoxy-6',6'-difluoro-5'-*O*-[(4,4'-dimethoxytriphenyl)methyl]-3',5'-propano-β-D-ribofuranosyl}thymine (9)

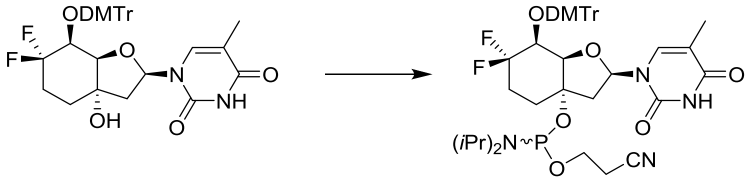

8 **9**

To a solution of the DMTr-protected nucleoside **8** (462 mg, 0.74 mmol) in dry THF (5 ml) was added DIPEA (0.52 ml, 2.99 mmol, 4.0 equiv) at rt. Afterwards CEP-Cl (0.33 ml, 1.48 mmol, 2.0 equiv) was added dropwise, and the resulting solution was stirred 3.1 h at rt. The solvent was then removed under reduced pressure and the residue was adsorbed on silica gel. Purification by CC (hex/EtOAc 1:1 + 0.5% Et_3N) yielded the phosphoramidite **9** (444 mg, 0.54 mmol, 73%) as a mixture of two isomers.

Data for **9**: R_f = 0.74 & 0.64 (hex/EtOAc 1:5); ^{1}H NMR (400 MHz, DMSO-d_6) δ 11.45 (*br*, 2H, 2x N*H*), 7.56 – 7.47 (*m*, 6H, 2x H-C(6), H-arom), 7.40 – 7.35 (*m*, 4H, H-arom), 7.34 – 7.28 (*m*, 8H, H-arom), 7.26 – 7.20 (*m*, 2H, H-arom), 6.93 – 6.87 (*m*, 8H, H-arom), 5.87 (*dd*, *J* = 8.5, 4.6 Hz, 1H, H-C(1')), 5.81 (*dd*, *J* = 8.5, 4.4 Hz, 1H, H-C(1')), 3.80 – 3.69 (*m*, 1H, H-C(5')), 3.74 (*s*, 12H, 4x *Me*O), 3.69 – 3.49 (*m*, 6H, H-C(4'), H-C(5'), 2x OCH_2CH$_2$CN), 3.48 – 3.34 (*m*, 5H, H-C(4'), 2x (Me$_2$C*H*)$_2$N), 2.71 – 2.62 (*m*, 4H, 2x OCH$_2$CH_2CN), 2.62 – 2.53 (*m*, 2H, H-C(2')), 2.24 – 2.14 (*m*, 2H, H-C(2')), 2.07 – 1.93 (*m*, 4H, H-C(7'), H-C(8')), 1.84 (*s*, 6H, 2x *Me*-C(5)), 1.82 – 1.53 (*m*, 4H, H-C(7'), H-C(8')), 1.09 (*dd*, *J* = 6.8, 2.8 Hz, 12H, (*Me*$_2$CH)$_2$N), 1.01 (*d*, *J* = 6.8 Hz, 4H, (*Me*$_2$CH)$_2$N), 0.95 (*d*, *J* = 6.7 Hz, 8H, (*Me*$_2$CH)$_2$N); ^{13}C NMR (101 MHz, DMSO-d_6) δ 163.6 (2x C(4)), 158.4, 158.4 (C-arom), 150.2, 150.2 (C(2)), 145.7, 145.5, 135.6, 135.3 (C-arom), 134.8 (*d*, *J* = 3.5 Hz, C(6)), 134.8 (*d*, *J* = 3.2 Hz, C(6)), 130.3, 130.3, 130.1, 127.8, 127.7, 127.6, 126.8, 126.8 (*C*H-arom), 121,2 (*dd*, *J* = 248.1, 245.3 Hz, C(6')), 121.0 (*dd*, *J* = 248.0, 245.0 Hz, C(6')), 118.9, 118.8 (*C*N), 113.0 (*C*H-arom), 109.3, 109.2 (C(5)), 87.8, 87.6 (*C*(Ph)$_3$), 83.0 (2x C(1')), 82.3 – 82.0 (*m*, C(4')), 82.1 – 81.8 (*m*, C(4')), 79.1 (*d*, *J* = 7.2 Hz, C(3')), 78.9 (*d*, *J* = 7.2 Hz, C(3')), 69.4 – 68.7 (*m*, 2x C(5')), 57.9 (*d*, *J* = 17.7 Hz, O*C*H$_2$CH$_2$CN), 57.6 (*d*, *J* = 18.2 Hz, O*C*H$_2$CH$_2$CN), 55.0 (*Me*O-DMTr), 42.7, 42.5 ((Me$_2$*C*H)$_2$N), 41.8 (*d*, *J* = 10.7 Hz, C(2')), 41.5 (*d*, *J* = 8.9 Hz, C(2')), 30.8 – 30.4 (*m*, C(8')), 30.3 – 30.0 (*m*, C(8')), 29.3 – 28.7 (*m*, 2x C(7')), 24.2, 24.1, 24.1, 24.0, 24.0 ((*Me*$_2$CH)$_2$N), 19.6 (2x *d*, *J* = 7.5 and 7.8 Hz, OCH$_2$*C*H$_2$CN), 12.5 (2x *Me*-C(5)); ^{19}F NMR (376 MHz, DMSO-d_6) δ -99.6 (*d*, *J* = 239.5 Hz, 1F), -100.1 (*d*, *J* = 239.7 Hz, 1F), -100.7 – -111.6 (*m*,

1F), -111.0 – -111.9 (*m*, 1F); ^{31}P NMR (162 MHz, DMSO-d_6) δ 143.1, 140.4; ESI$^+$-HRMS *m/z* calcd for $C_{43}H_{51}O_8N_4F_2NaP$ [(M + Na)$^+$] 843.3305, found 843.3280.

(1*S*,4*S*,6*R*,8*S*)-3,3-Difluoro-4-iodo-8-methoxy-6-[(trimethylsilyl)oxy]-9-oxabicyclo-[4.3.0]non-2-one (11) and (1*S*,6*R*,8*S*)-3,3-difluoro-2,2-dihydroxy-4-iodo-8-methoxy-6-[(trimethylsilyl)oxy]-9-oxabicyclo[4.3.0]nonane (12)

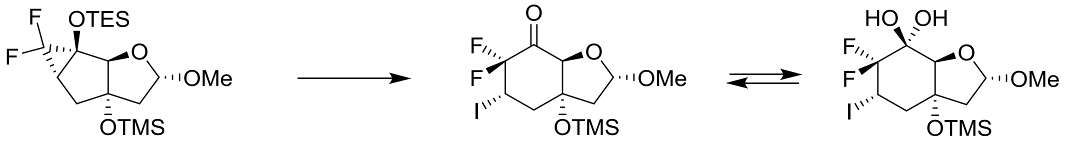

10 **11** **12**

To a solution of the tricyclic sugar **10** (102 mg, 0.25 mmol) in dry DCM (1.4 ml) at 0 °C was added NIS (85 mg, 0.38 mmol, 1.5 equiv). The mixture was stirred 20 min at 0 °C followed by 2.25 h at rt, before it was diluted with Et_2O (10 ml). Then it was washed with sat. $Na_2O_3S_2$ sol. (2 × 10 ml) and sat. $NaHCO_3$ sol. (10 ml). Afterwards the organic phase was extracted with Et_2O (3 × 30 ml), dried ($MgSO_4$) and concentrated. Purification by CC (hex/Et_2O 1:1) gave the inseparable mixture of **11/12** (64 mg, 0.15 mmol, 60%) as colourless oil.

Data for **11/12**: R_f = 0.20 (hex/Et_2O 1:1); ^{1}H NMR (400 MHz, $CDCl_3$) δ 5.28 (*d*, *J* = 6.2 Hz, 1H, H-C(8)), 5.24 (*dd*, *J* = 6.5, 1.2 Hz, 1H, H-C(8)), 4.90 (*dd*, *J* = 5.6, 1.5 Hz, 1H, H-C(1)), 4.38 – 4.24 (*m*, 1H, H-C(4)), 4.00 – 3.86 (*m*, 2H, H-C(1), H-C(4)), 3.80 (*d*, *J* = 3.0 Hz, 1H, *H*O-C(2)), 3.78 (*s*, 1H, *H*O-C(2)), 3.43 (*s*, 3H, *Me*O), 3.40 (*s*, 3H, *Me*O), 2.80 – 2.74 (*m*, 3H, H-C(5)), 2.51 (*t*, *J* = 13.8 Hz, 1H, H-C(5)), 2.24 (*dd*, *J* = 13.7, 6.3 Hz, 1H, H-C(7)), 2.04 – 1.92 (*m*, 2H, H-C(7)), 1.83 (*dd*, *J* = 14.3, 6.5 Hz, 1H, H-C(7)), 0.20 (*s*, 9H, Me_3Si), 0.15 (*s*, 9H, Me_3Si); ^{13}C NMR (101 MHz, $CDCl_3$) δ 191.5 (*dd*, *J* = 30.5, 22.4 Hz, C(2)), 117.4 (*dd*, *J* = 256.8, 246.2 Hz, C(3)), 114.1 (*dd*, *J* = 263.3, 249.5 Hz, C(3)), 107.0 (C(8)), 105.3 (C(8)), 91.2 (*dd*, *J* = 29.9, 21.1 Hz, (C(2)), 89.7 (*t*, *J* = 2.9 Hz, C(1)), 89.2 (*d*, *J* = 3.4 Hz, C(1)), 83.6 (C(6)), 81.0 (*d*, J = 0.8 Hz, C(6)), 56.1 (*Me*O), 55.8 (*Me*O), 43.8 (C(7)), 43.6 (*dd*, *J* = 4.7, 2.1 Hz, C(5)), 42.8 (*dd*, *J* = 4.7, 1.7 Hz, C(5)), 42.4 (C(7)), 17.8 (*t*, *J* = 23.5 Hz, C(4)), 17.2 (*dd*, *J* = 25.0, 23.0 Hz, C(4)), 2.1 (Me_3Si), 2.0 (Me_3Si); ^{19}F NMR (376 MHz, $CDCl_3$) δ -108.1 (*d*, *J* = 241.0 Hz, 1F), -113.0 (*dd*, *J* = 243.0, 3.7 Hz, 1F), -113.8 (*ddd*, *J* = 241.0, 27.4, 5.8 Hz, 1F), -

119.3 (*dd*, *J* = 243.0, 26.3 Hz, 1F); ESI$^+$-HRMS *m/z* calcd for $C_{12}H_{20}O_4F_2ISi$ [(M + H)$^+$] 421.0138, found 421.0127; ESI$^+$-HRMS *m/z* calcd for $C_{12}H_{21}O_5F_2INaSi$ [(M + Na)$^+$] 461.0063, found 461.0052.

(1*S* and *R*,6*R*,8*S*)-3,3-Difluoro-2-hydroxy-8-methoxy-6-[(trimethylsilyl)oxy]-9-oxabicyclo[4.3.0]non-2-one (13)

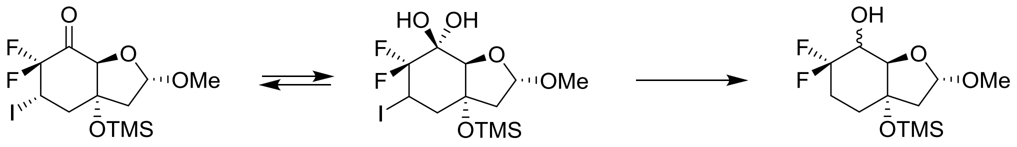

11 **12** **13**

To a solution of the sugars **11/12** (51 mg, 0.12 mmol) in dry toluene (1.2 ml) was added AIBN (2 mg, 0.01 mmol, 0.1 equiv) and Bu_3SnH (0.08 ml, 0.30 mmol, 2.6 equiv). The mixture was then heated up to 90 °C and stirred for 30 min at this temperature. After cooling down to rt, it was evaporated to dryness. The crude was purified by CC (hex/Et_2O 10:1 → 1:5) yielding the bicyclic sugars **13** (22 mg, 0.07 mmol, 62%) as colourless oil.

Data for **13**: R_f = 0.46 (hex/Et_2O 1:5); ^{1}H NMR (400 MHz, $CDCl_3$) δ 4.96 (*dd*, *J* = 5.9, 4.4 Hz, 1H, H-C(8)), 4.98 – 4.93 (*m*, 1H, H-C(8)), 4.13 (*tt*, *J* = 4.6, 1.8 Hz, 1H, H-C(1)), 4.06 – 4.03 (*m*, 1H, H-C(1)), 3.99 – 3.89 (*m*, 1H, H-C(2)), 3.89 (*dddd*, *J* = 21.7, 11.0, 6.6, 4.5 Hz, 1H, H-C(2)), 3.40 (*s*, 3H, *Me*O), 3.38 (*s*, 3H, *Me*O), 2.87 (*d*, *J* = 8.7 Hz, 1H, *H*O-C(2)), 2.51 (*dd*, *J* = 10.8, 1.5 Hz, 1H, *H*O-C(2)), 2.28 (*dd*, *J* = 13.0, 5.9 Hz, 1H, H-C(7)), 2.26 – 2.20 (*m*, 1H, H-C(7)), 2.10 – 1.93 (*m*, 3H, H-C(7), H-C(4)), 2.03 (*dd*, *J* = 13.0, 4.4 Hz, 1H, H-C(7)), 1.85 – 1.64 (*m*, 6H, H-C(4), H-C(5)); ^{13}C NMR (101 MHz, $CDCl_3$) δ 121.6 (*dd*, *J* = 247.8, 244.6 Hz, C(3)), 121.5 (*dd*, *J* = 246.6, 243,7 Hz, C(3)), 104.1, 103.8 (C(8)), 81.1 (*dd*, *J* = 9.4, 1.7 Hz, C(1)), 80.8 (*d*, *J* = 5.6 Hz, C(1)), 79.0 (*d*, *J* = 1.6 Hz, C(6)), 78.1 (*d*, *J* = 0.7 Hz, C(6)), 68.8 (*dd*, *J* = 23.4, 20.6 Hz, C(2)), 68.9 – 68.2 (*m*, C(2)), 55.9, 55.8 (*Me*O), 46.7 (*d*, *J* = 1.5 Hz, C(7)), 45.9 (C(7)), 32.0 (*dd*, *J* = 8.9, 1.1 Hz, C(5)), 31.7 (*d*, *J* = 9.4 Hz, C(5)), 28.6 (*dd*, *J* = 23.9, 22.7 Hz, C(4)), 24.9 (*t*, *J* = 23.6 Hz, C(4)), 2.0 (*Me*$_3$Si), 1.9 (*Me*$_3$Si); ^{19}F NMR (376 MHz, $CDCl_3$) δ -103.8 (*d*, *J* = 240.4 Hz, 1F), -104.6 – -104.8 (*m*, 1F), -105.5 – -106.4 (*m*, 1F), -116.3 – -117.3 (*m*, 1F); ESI$^+$-HRMS *m/z* calcd for $C_{12}H_{22}O_4F_2NaSi$ [(M + Na)$^+$] 319.1148, found 319.1150.

4. NMR spectra of the new compounds

1-[(2’*R* and *S*,3’*R*,5’*R*,6’*S*)-2’-deoxy-3’,5’-ethano-5’-*O*-triethylsilyl-8’,8-difluoro-2’-iodo-5’,6’-methano-3’-*O*-trimethylsilyl-α and β-D-ribofuranosyl]thymine (2α/β)

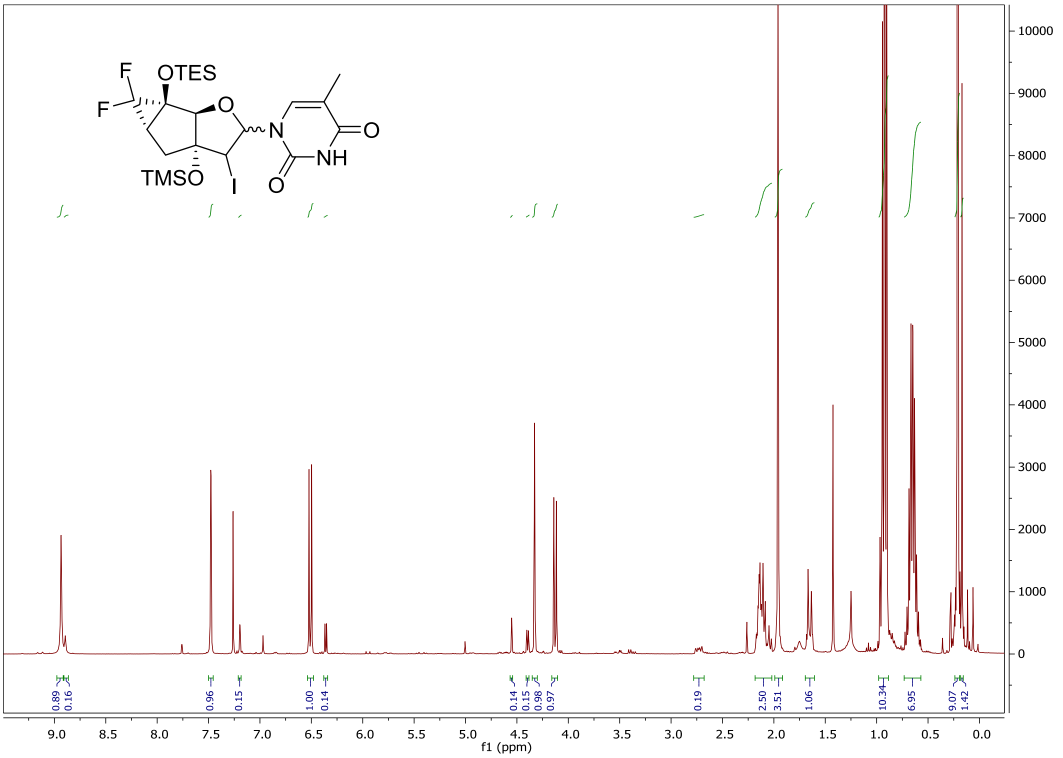

Figure S1: ^{1}H NMR (400 MHz, $CDCl_3$) spectra of **2α/β**.

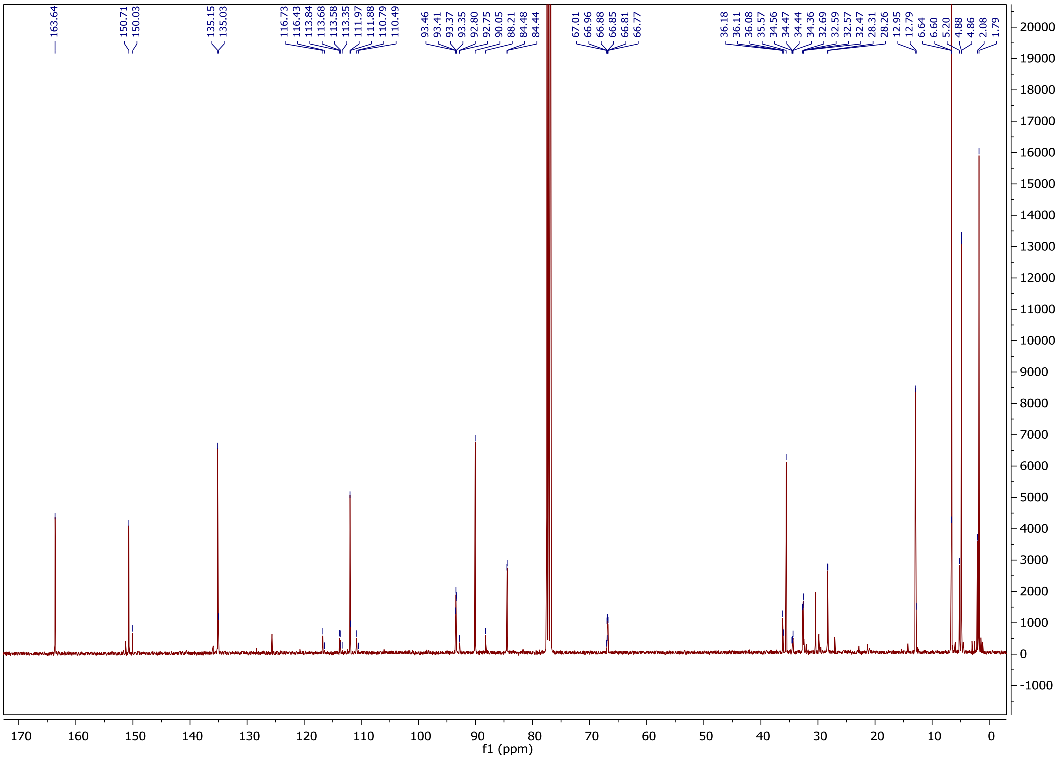

Figure S2: ^{13}C NMR (101 MHz, $CDCl_3$) spectra of **2α/β**.

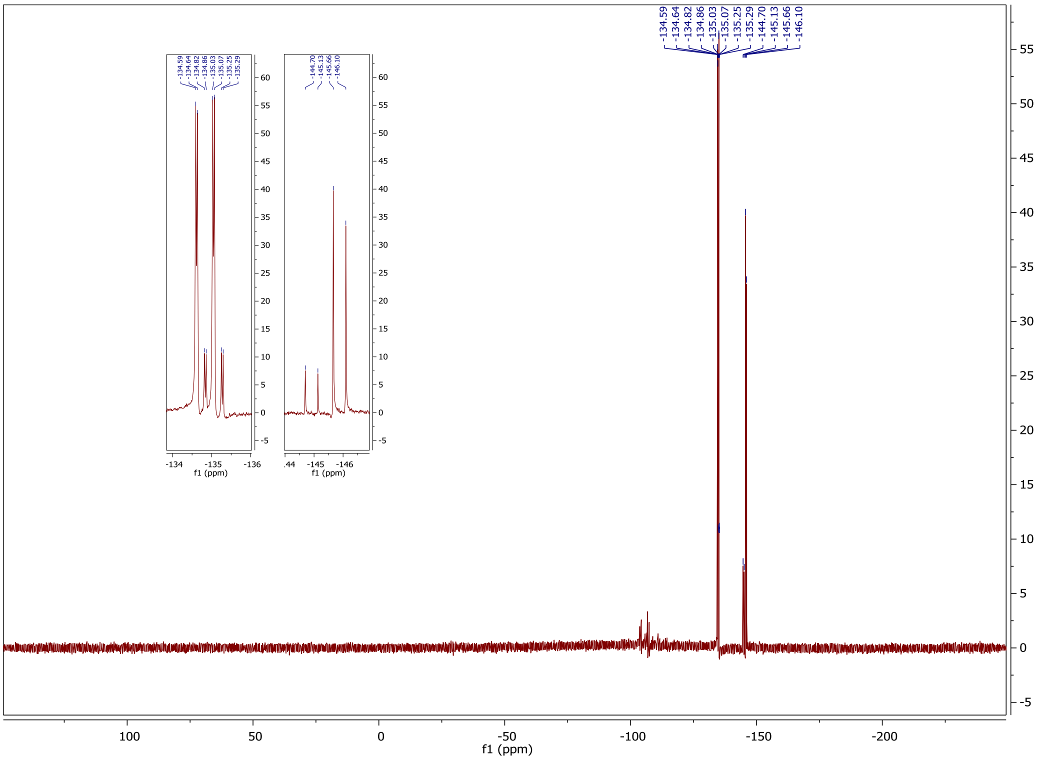

Figure S3: ^{19}F NMR (376 MHz, $CDCl_3$) spectra of **2α/β**.

1-[(2’*R*,3’*R*,7’*R*)-2’-Deoxy-6’6’-difluoro-2’,7’-diiodo-3’-*O*-trimethylsilyl-3’,5’-propano-5’-oxo-β-D-ribofuranosyl]thymine (3)

and

1-[(2’*R*,3’*R*,7’*R*)-2’-deoxy-6’,6’-difluoro-5’,5’-dihydroxy-2’,7’-diiodo-3’-*O*-trimethylsilyl-3’,5’-propano-β-D-ribofuranosyl]thymine (4)

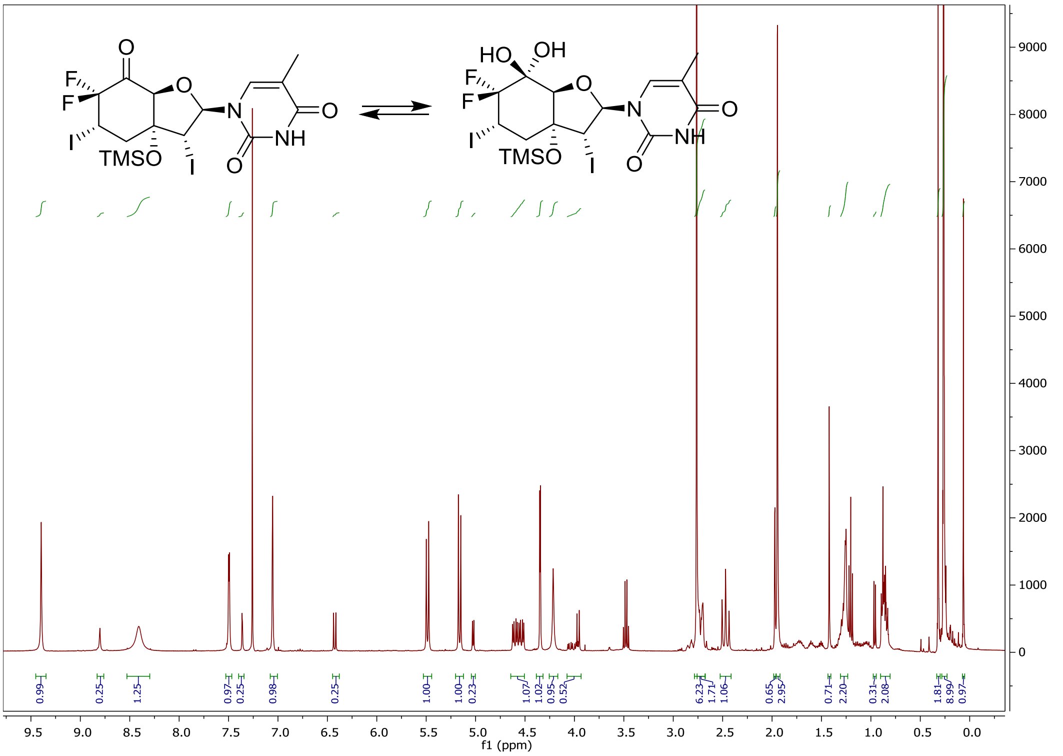

Figure S4: ^{1}H NMR (400 MHz, $CDCl_3$) spectra of **3/4**.

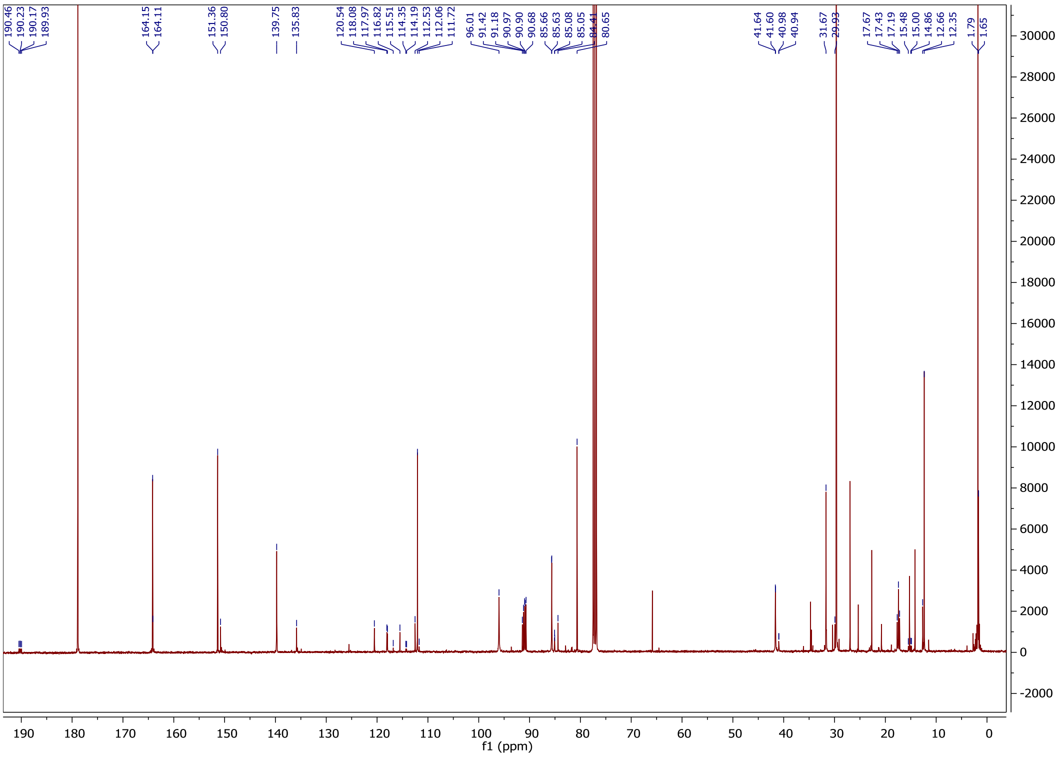

Figure S5: ^{13}C NMR (101 MHz, $CDCl_3$) spectra of **3/4**.

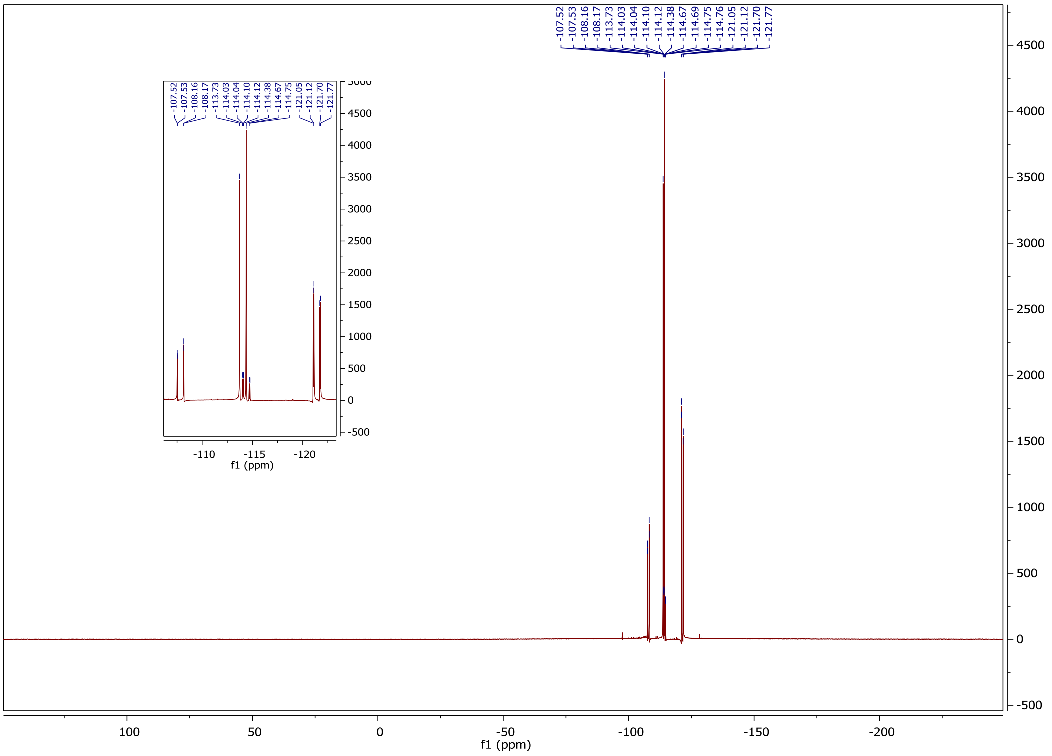

Figure S6: ^{19}F NMR (376 MHz, $CDCl_3$) spectra of **3/4**.

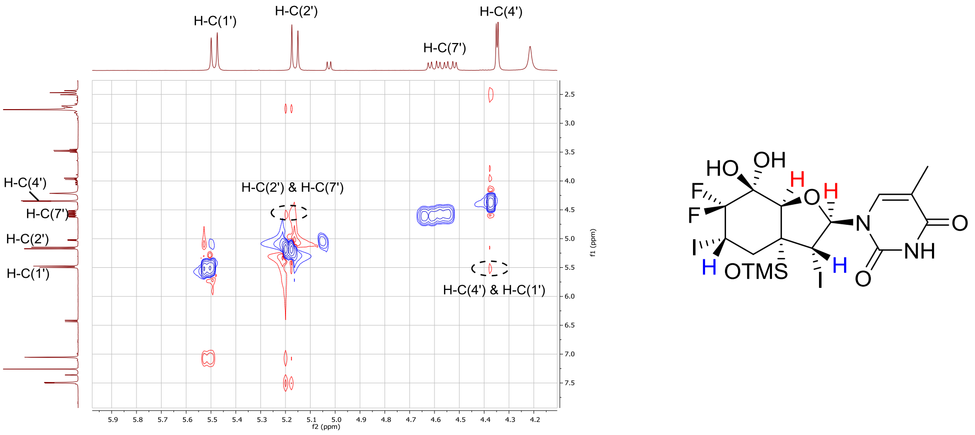

Figure S7: $^1H/^1H$-ROESY spectrum (400 MHz, $CDCl_3$) of **4**.

1-[(3’*S*,5’*S*)-2’-Deoxy-6’,6’-difluoro-3’-*O*-trimetylsilyl-3’,5’-propano-β-D-ribofuranosyl]thymine (6)

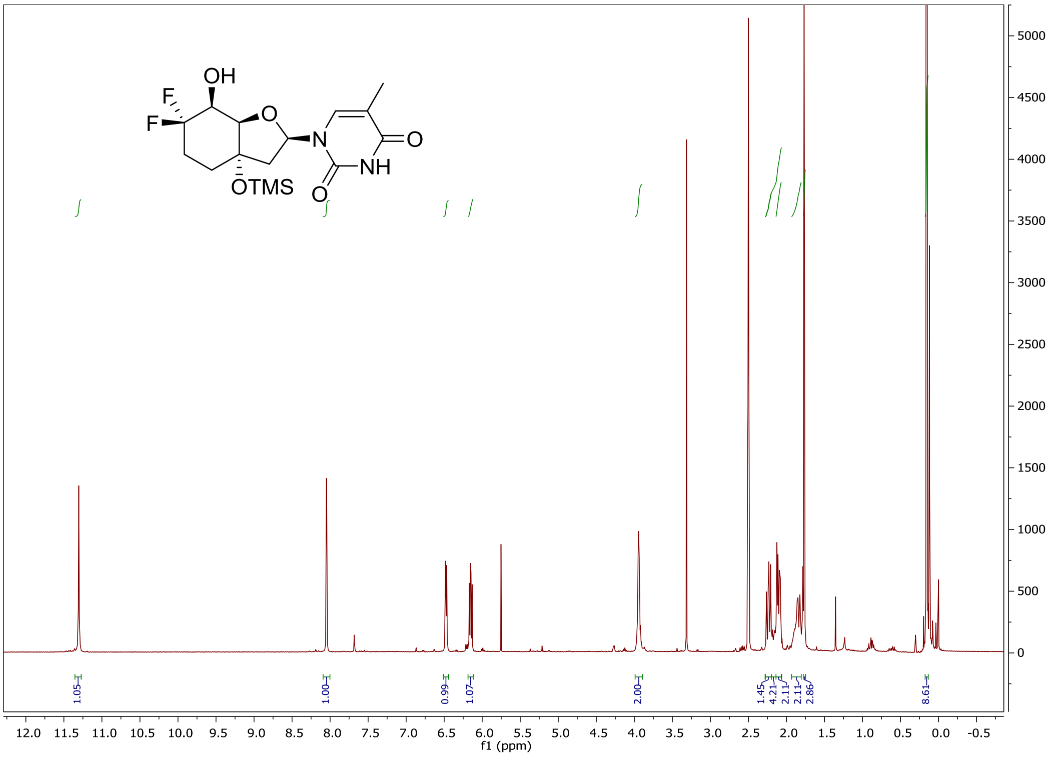

Figure S8: ^{1}H NMR (400 MHz, DMSO-d_6) spectra of **6**.

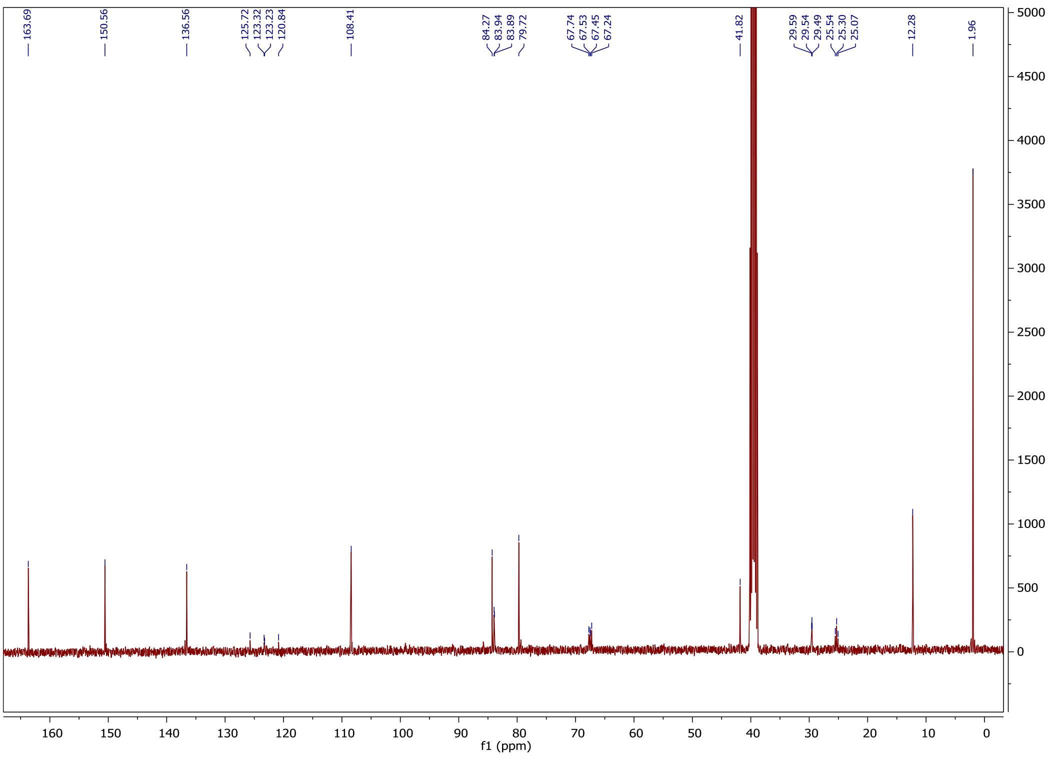

Figure S9: ^{13}C NMR (101 MHz, DMSO-d_6) spectra of **6**.

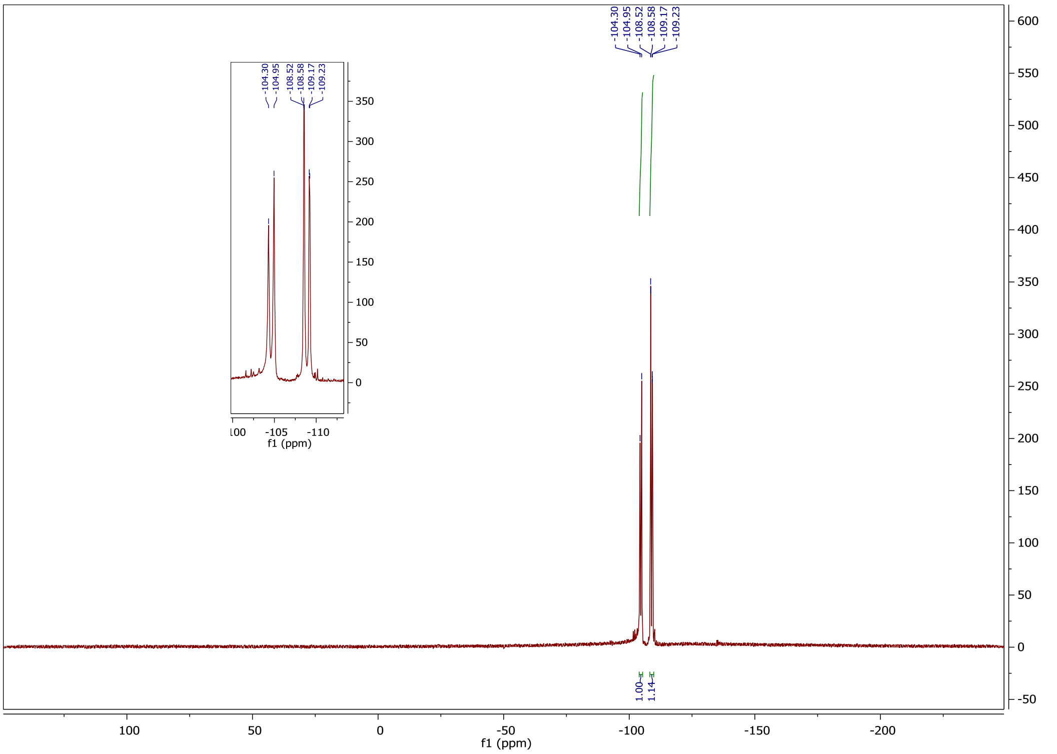

Figure S10: ^{19}F NMR (376 MHz, DMSO-d_6) spectra of **6**.

1-[(3’*S*,5’*S*)-2’-Deoxy-6’,6’-difluoro-3’,5’-propano-β-D-ribofuranosyl]thymine (7)

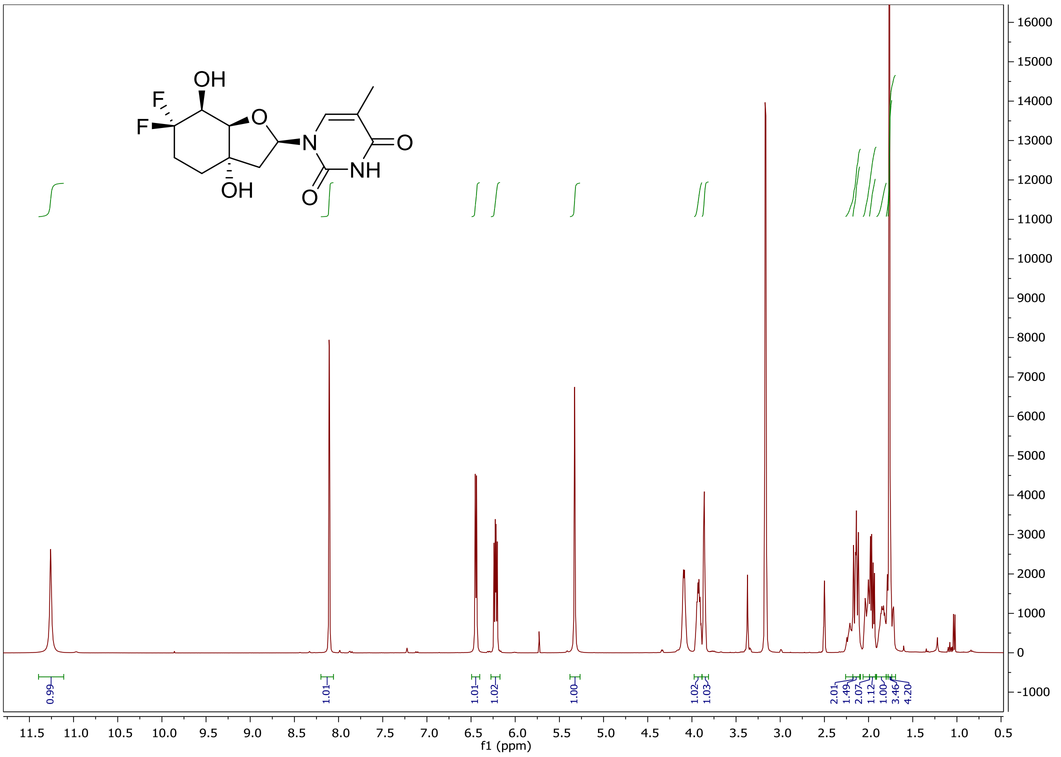

Figure S11: ^{1}H NMR (400 MHz, DMSO-d_6) spectra of **7**.

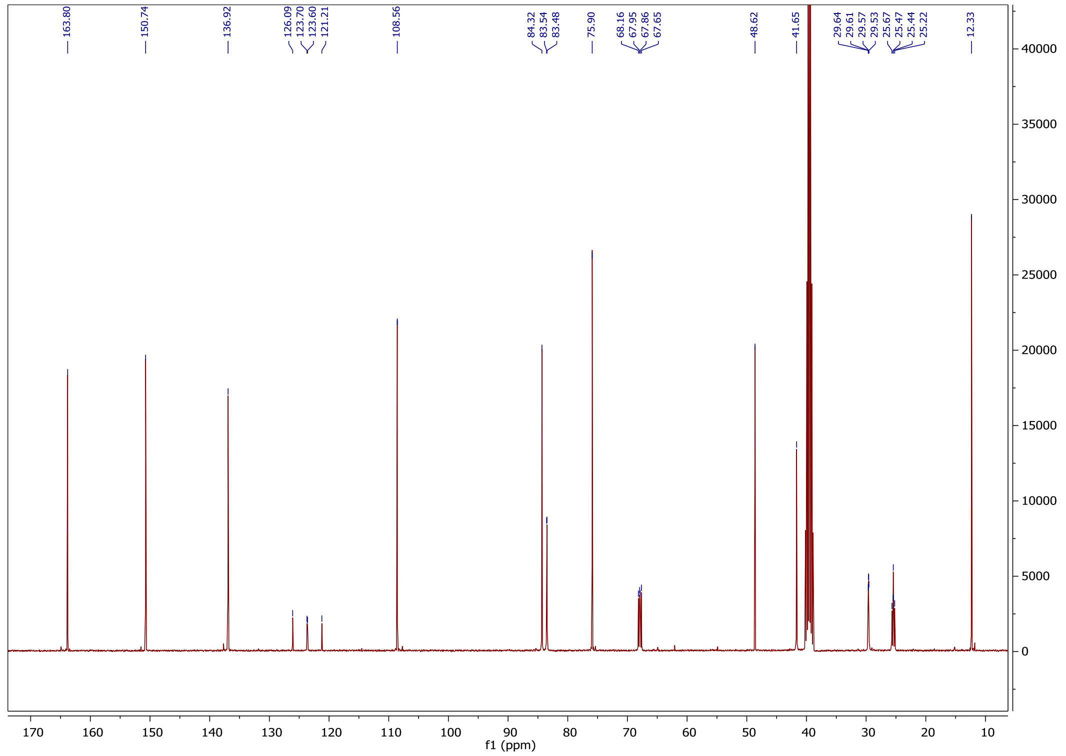

Figure S12: ^{13}C NMR (101 MHz, DMSO-d_6) spectra of **7**.

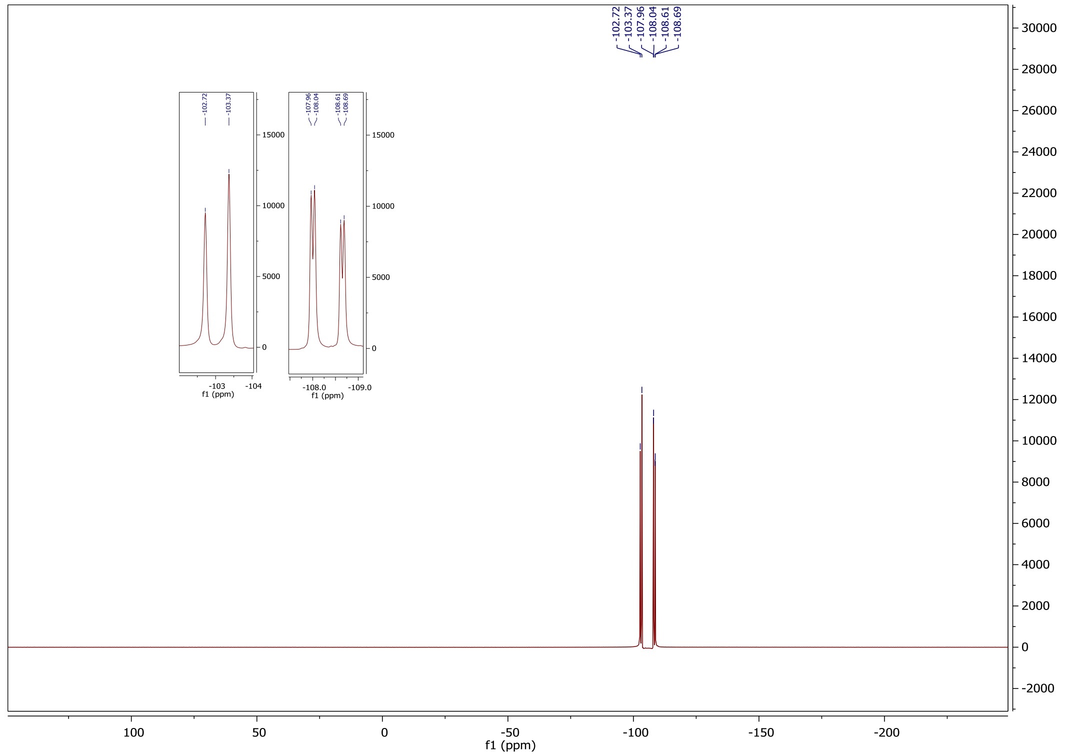

Figure S13: ^{19}F NMR (376 MHz, DMSO-d_6) spectra of **7**.

1-{(3'*S*,5'*S*)-2'-Deoxy-6',6'-difluoro-5'-*O*-[(4,4'-dimethyoxytriphenyl)methyl]-3',5'-propano-β-D-ribofuranosyl}thymine (8)

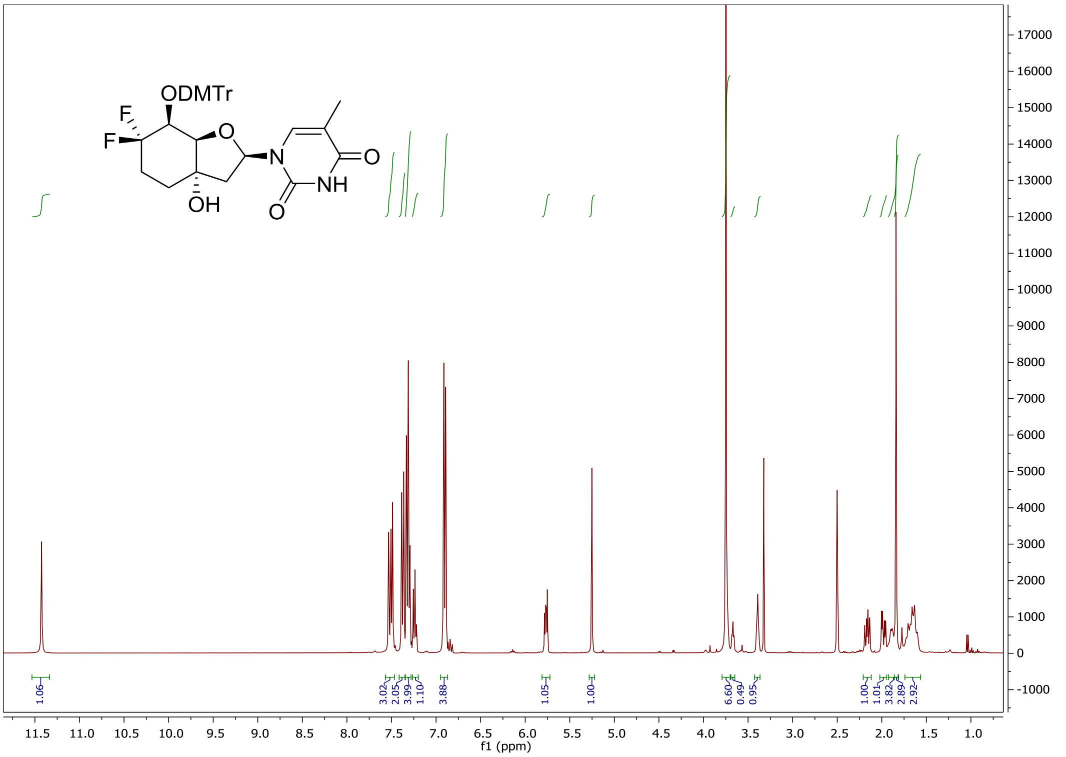

Figure S14: ^{1}H NMR (400 MHz, DMSO-d_6) spectra of **8**.

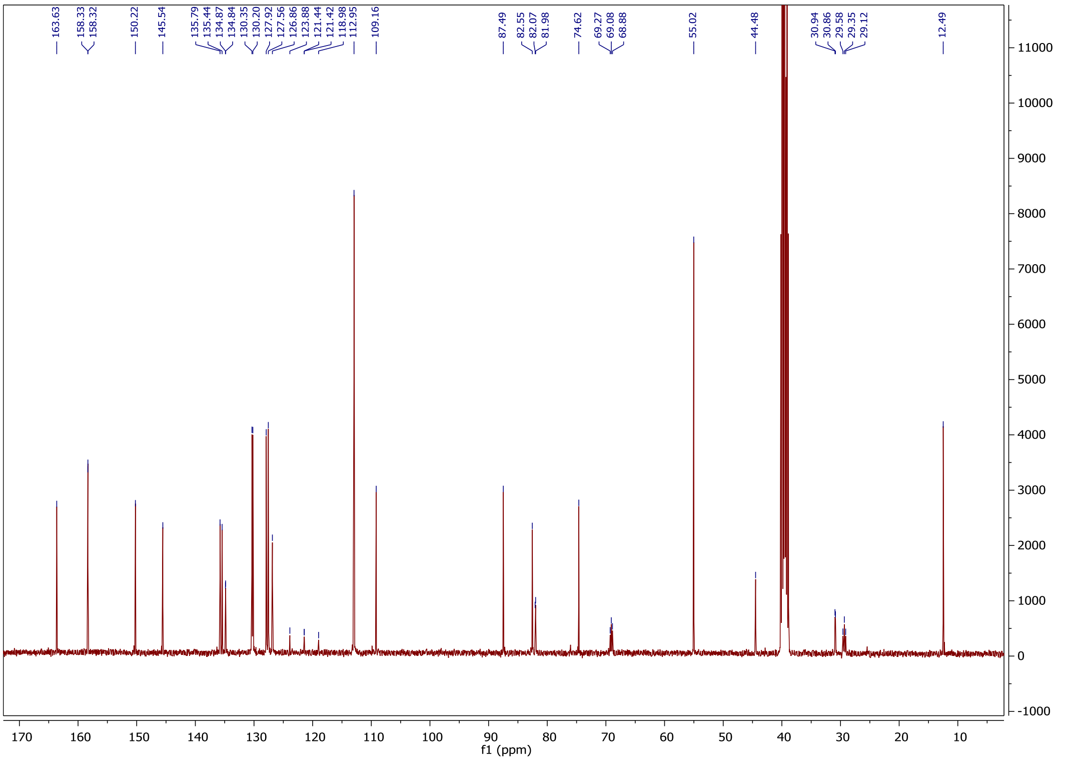

Figure S15: ^{13}C NMR (101 MHz, DMSO-d_6) spectra of **8**.

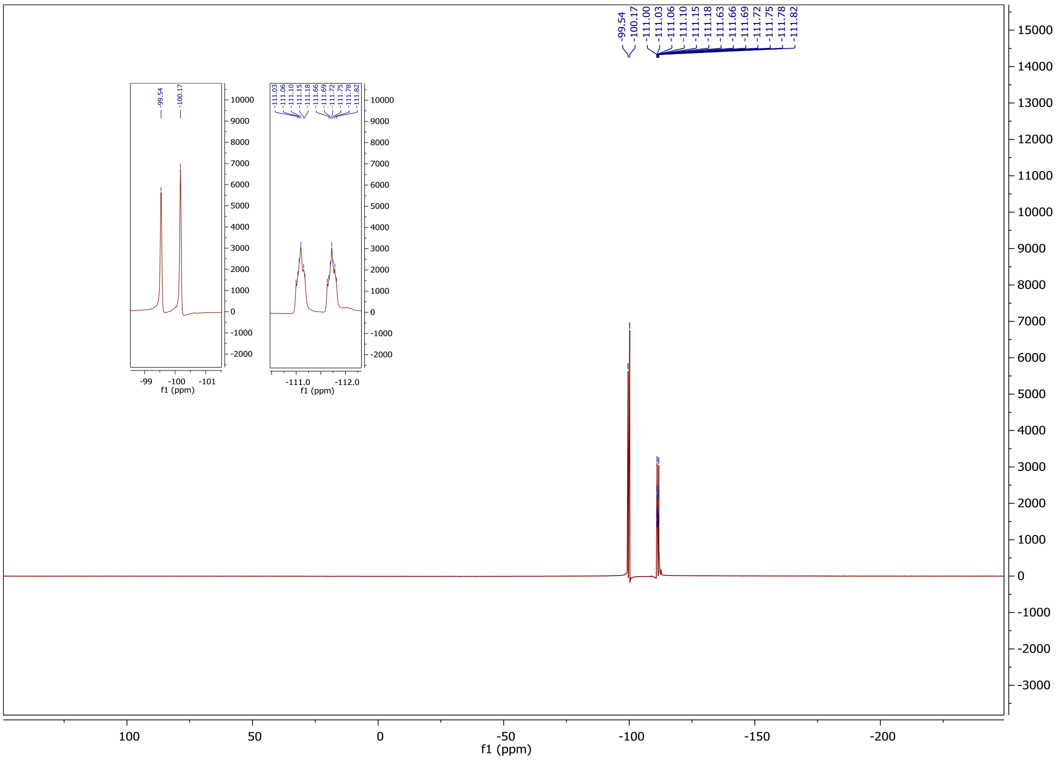

Figure S16: ^{19}F NMR (376 MHz, DMSO-d_6) spectra of **8**.

1-{(3’*S*,5’*S*)-3’-*O*-[(2-Cyanoethoxy)diisopropylaminophosphanyl]-2’-deoxy-6’,6’-difluoro-5’-*O*-[(4,4’-dimethoxytriphenyl)methyl]-3’,5’-propano-β-D-ribofuranosyl}thymine (9)

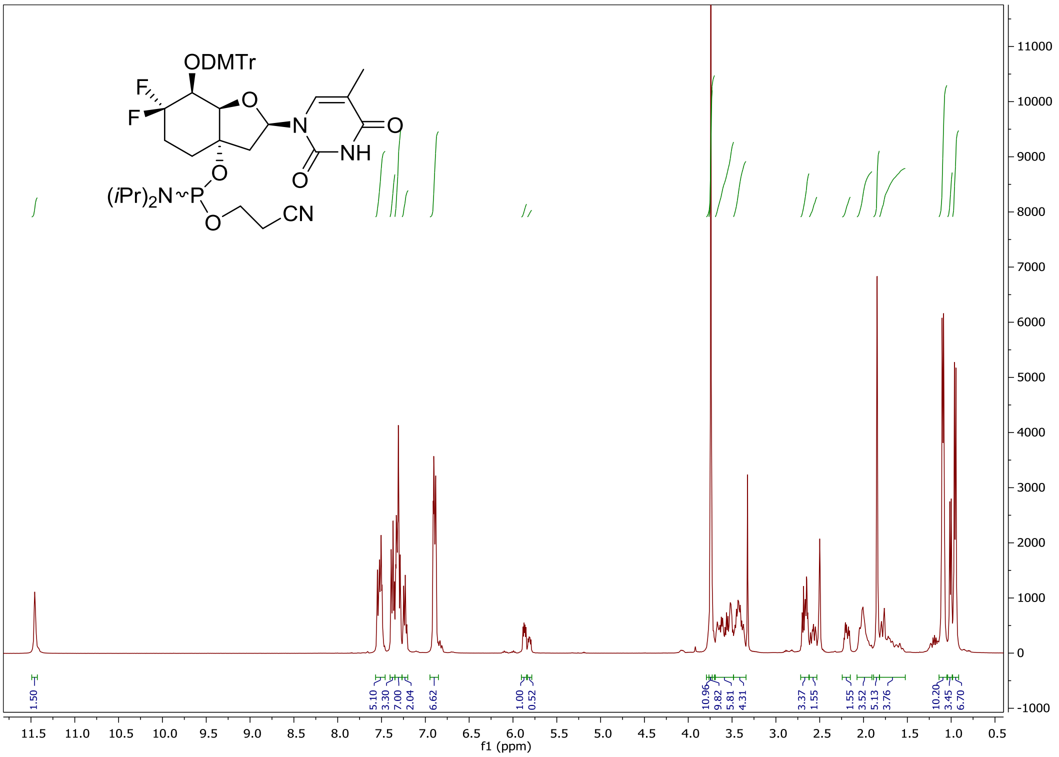

Figure S17: ^{1}H NMR (400 MHz, DMSO-d_6) spectra of **9**.

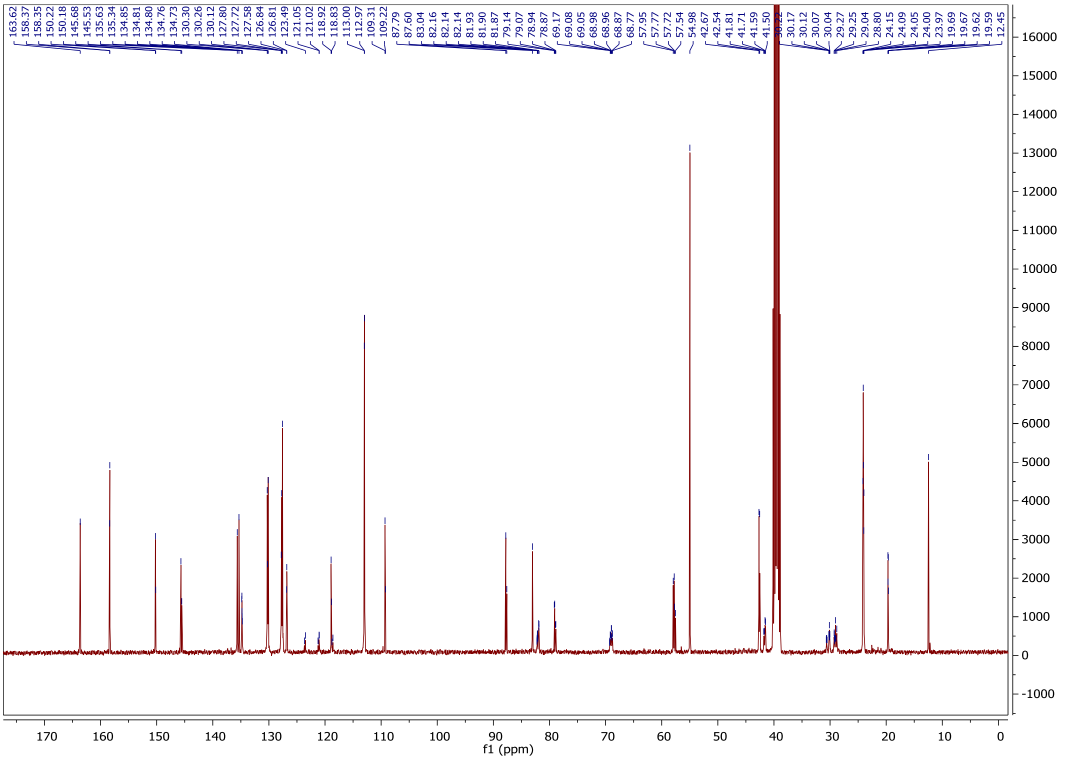

Figure S18: ^{13}C NMR ((101 MHz, DMSO-d_6) spectra of **9**.

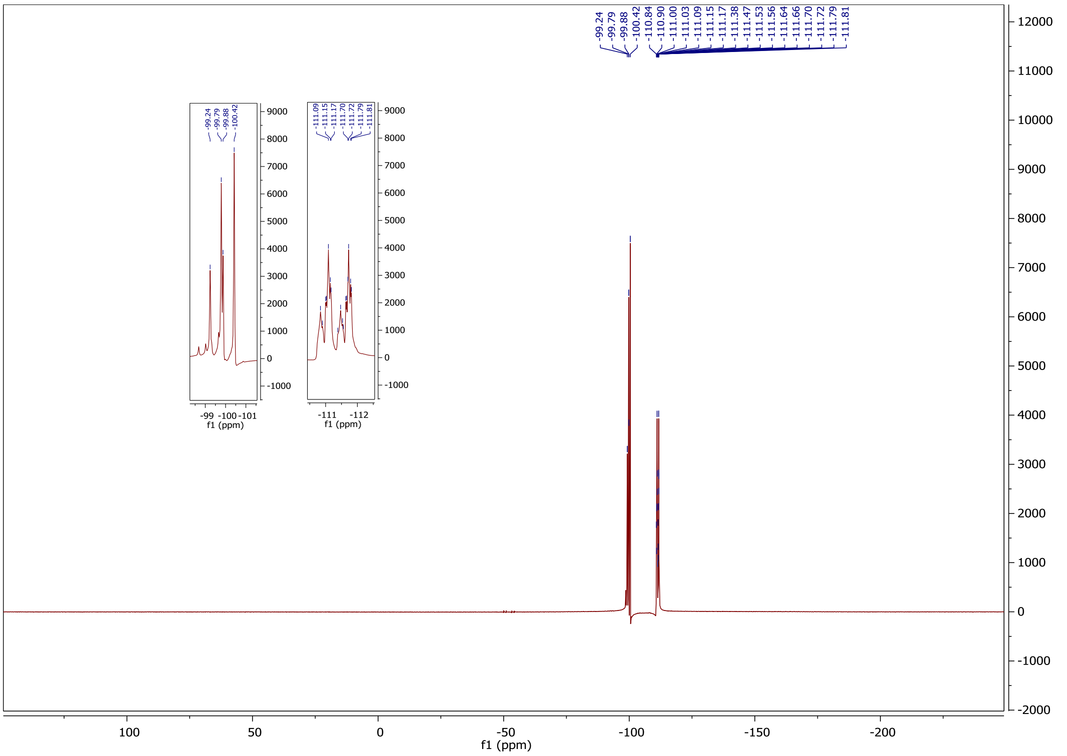

Figure S19: ^{19}F NMR (376 MHz, DMSO-d_6) spectra of **9**.

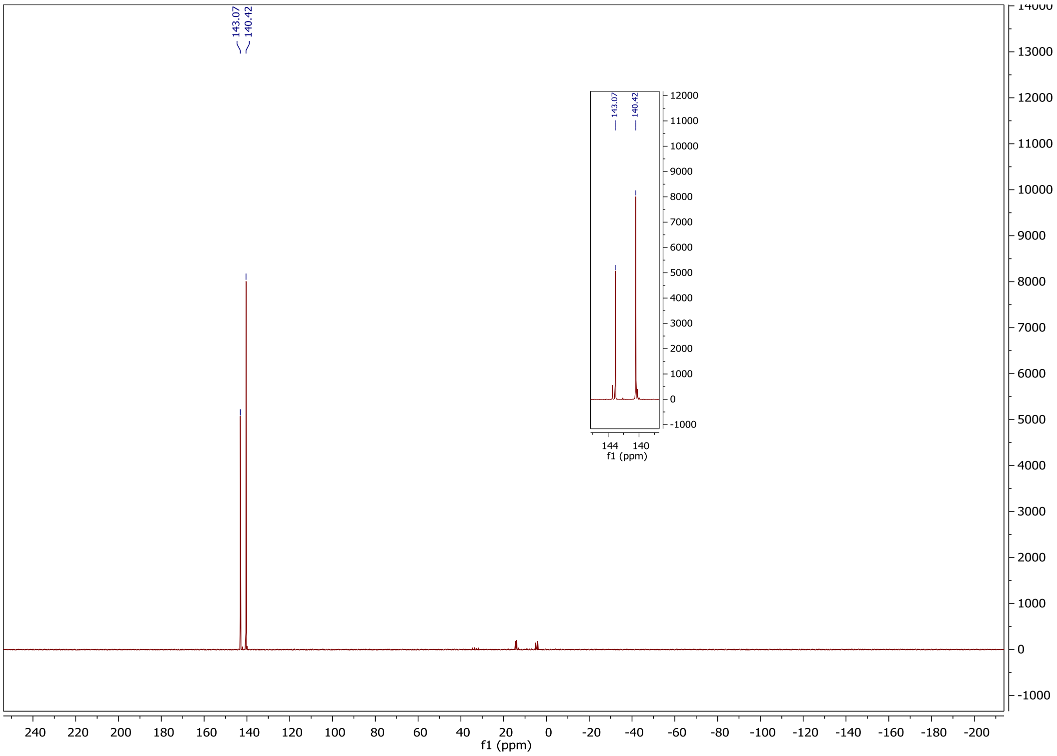

Figure S20: ^{31}P NMR (162 MHz, DMSO-d_6) spectra of **9**.

(1*S*,4*S*,6*R*,8*S*)-3,3-Difluoro-4-iodo-8-methoxy-6-[(trimethylsilyl)oxy]-9-oxabicyclo-[4.3.0]non-2-one (11)

and

(1*S*,6*R*,8*S*)-3,3-difluoro-2,2-dihydroxy-4-iodo-8-methoxy-6-[(trimethylsilyl)oxy]-9-oxabicyclo[4.3.0]nonane (12)

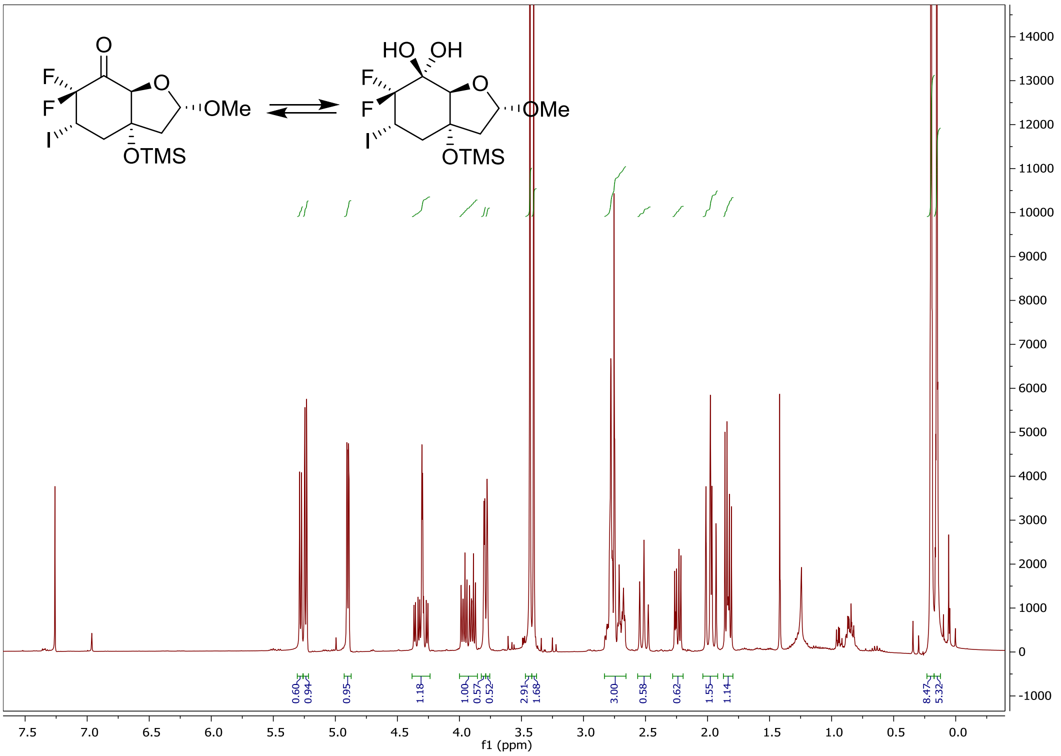

Figure S21: ^{1}H NMR (400 MHz, $CDCl_3$) spectra of **11/12**.

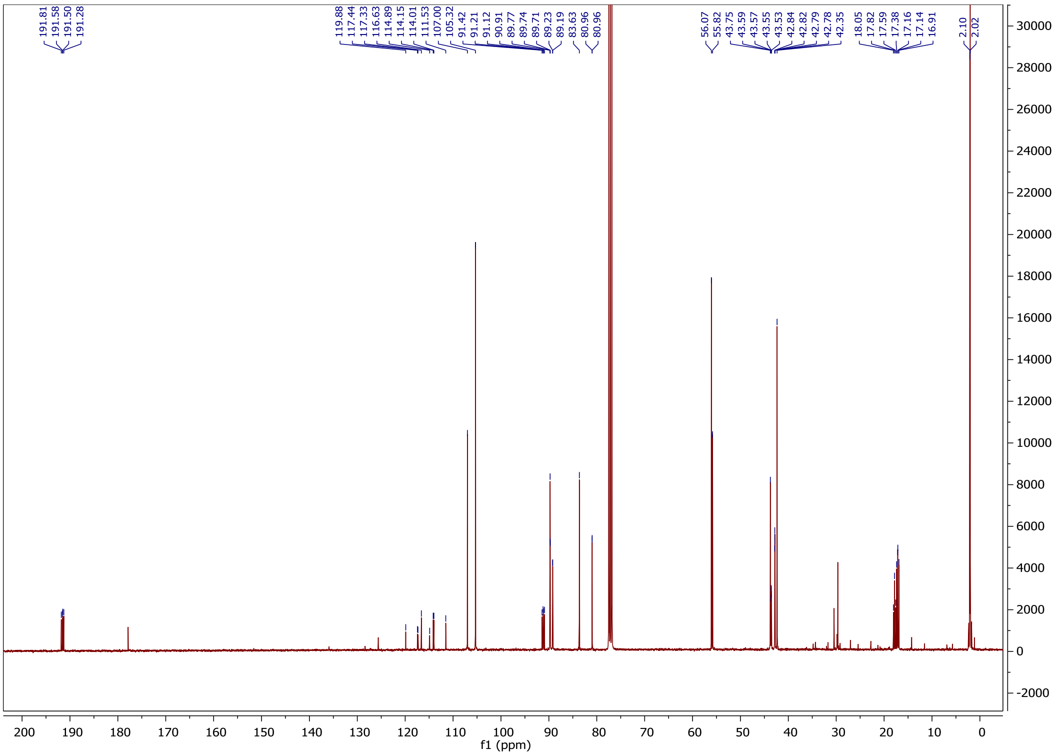

Figure S22: ^{13}C NMR (101 MHz, $CDCl_3$) spectra of **11/12**.

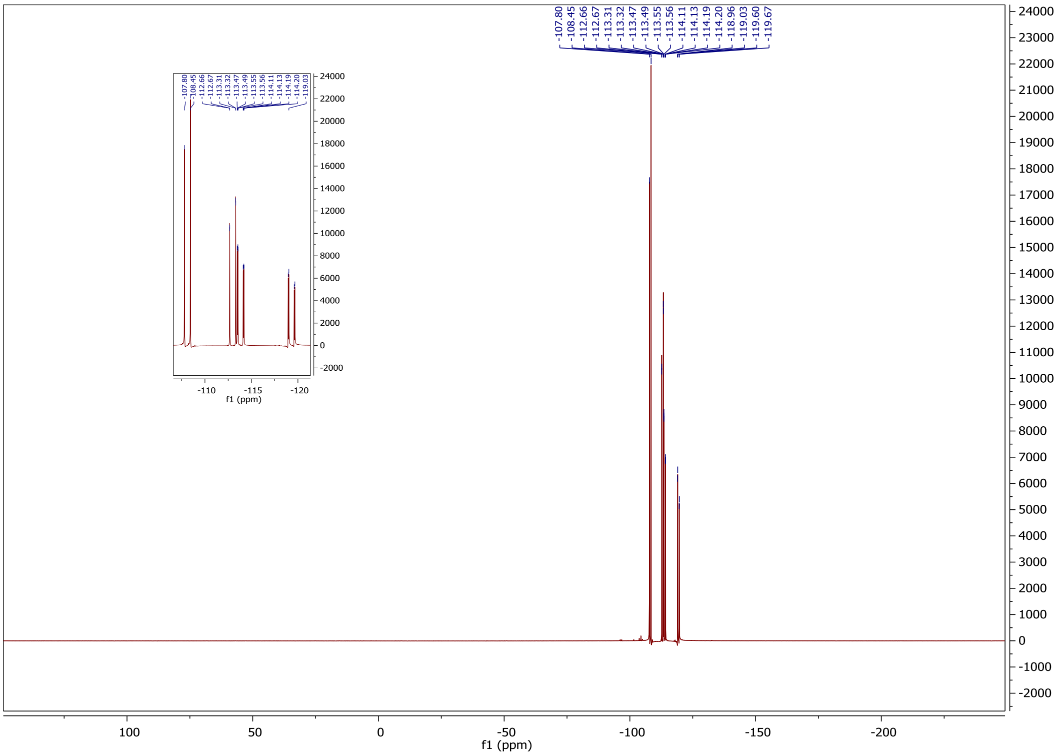

Figure S23: ^{19}F NMR (376 MHz, $CDCl_3$) spectra of **11/12**.

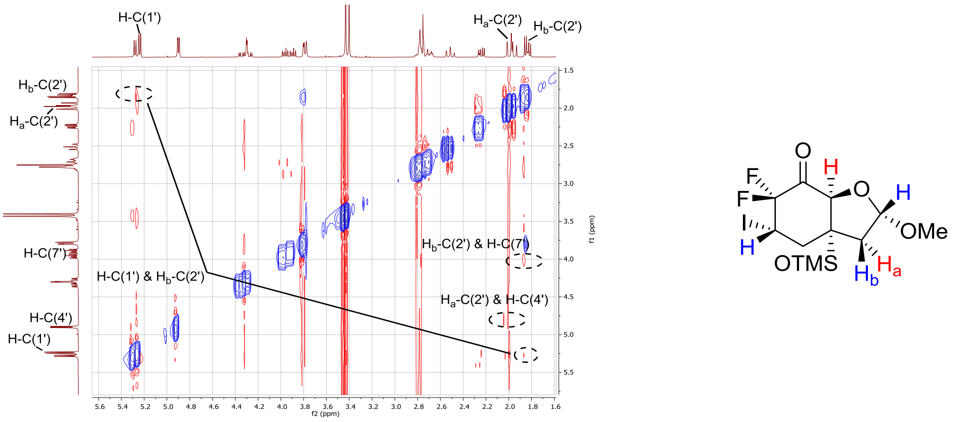

Figure S24: $^{1}H/^{1}H$-ROESY spectrum (400 MHz, $CDCl_3$) of **11**.

(1*S* and *R*,6*R*,8*S*)-3,3-Difluoro-2-hydroxy-8-methoxy-6-[(trimethylsilyl)oxy]-9-oxabicyclo[4.3.0]non-2-one (13)

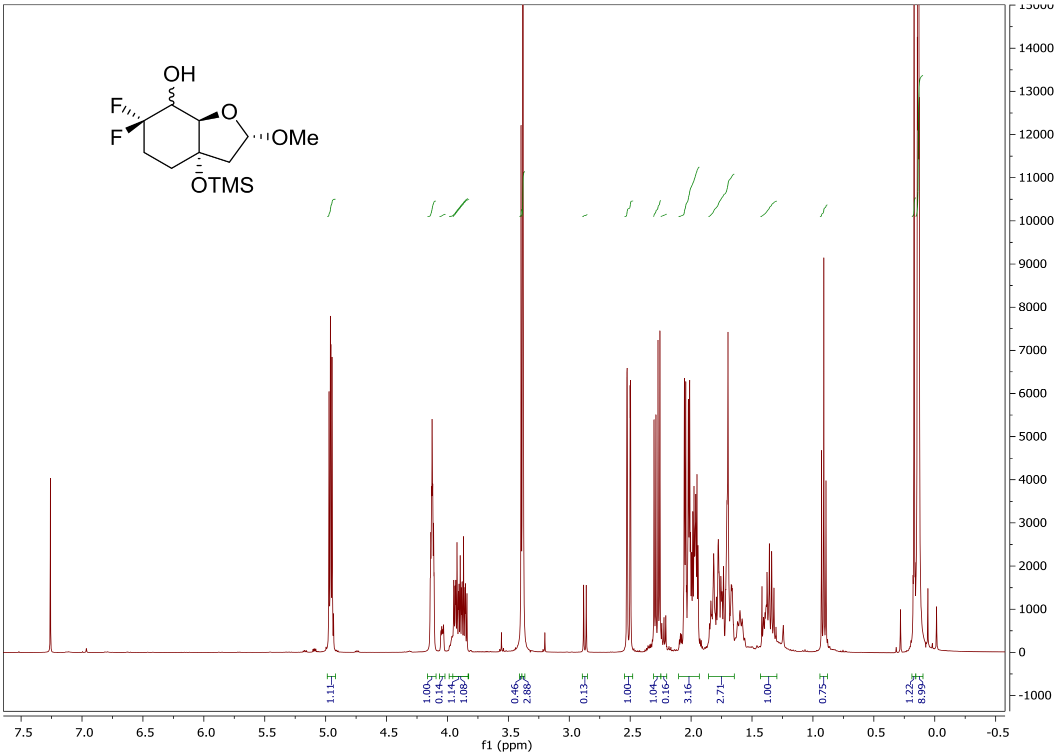

Figure S25: ^{1}H NMR (400 MHz, $CDCl_3$) spectra of **13**.

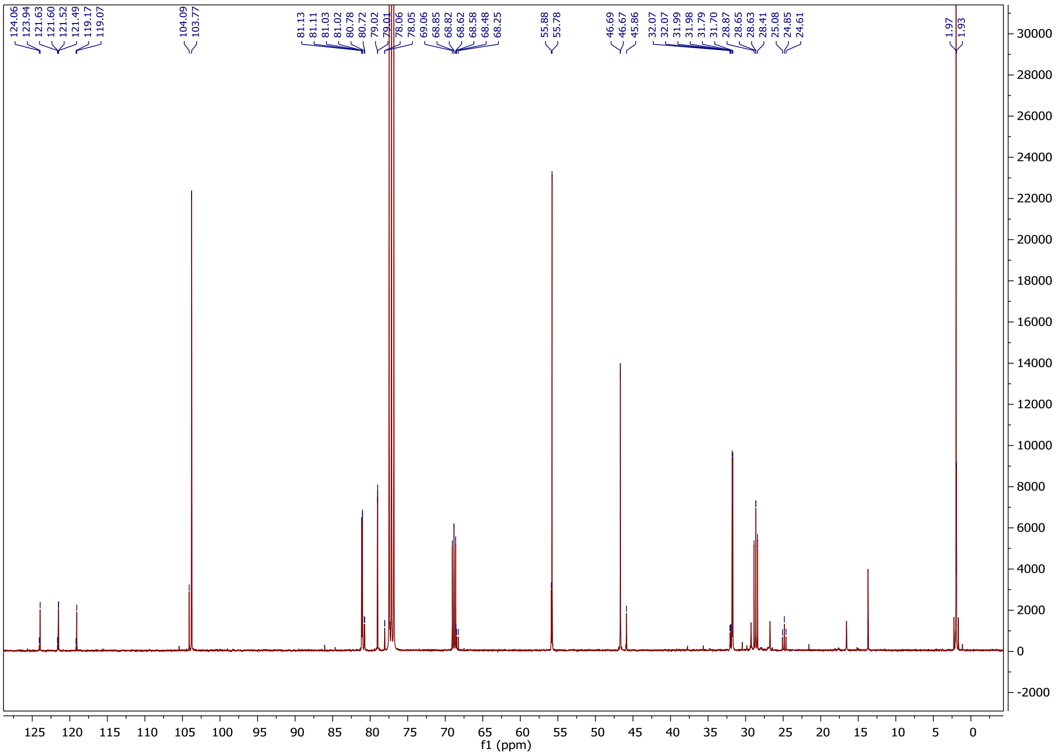

Figure S26: ^{13}C NMR (101 MHz, $CDCl_3$) spectra of **13**.

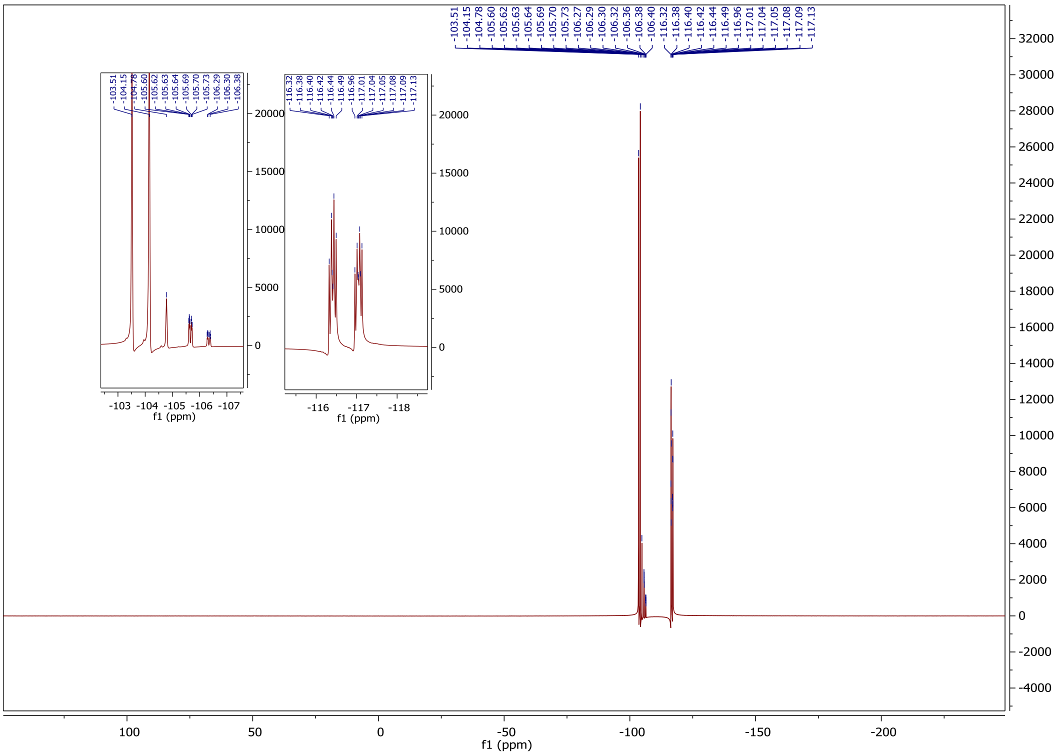

Figure S27: ^{19}F NMR (376 MHz, $CDCl_3$) spectra of **13**.

5. X-ray crystal structure analysis

Crystal-structure determination of 1-[(3'*S*,5'*S*)-2'-deoxy-6',6'-difluoro-3'-*O*-trimetylsilyl-3',5'-propano-β-D-ribofuranosyl]thymine (6)

A crystal of $C_{16}H_{24}F_2N_2O_5Si$ was mounted in air at ambient conditions. All measurements were made on an Oxford Diffraction SuperNova area-detector diffractometer [5] using mirror optics monochromated Mo Kα radiation (λ = 0.71073 Å) and Al filtered [6]. The unit cell constants and an orientation matrix for data collection were obtained from a least-squares refinement of the setting angles of reflections in the range 1.6 < θ < 26.7°. A total of 535 frames were collected using ω scans, with 35 + 35 seconds exposure time, a rotation angle of 1.0° per frame, a crystal-detector distance of 65.0 mm, at *T* = 173(2) K.

Data reduction was performed using the CrysAlisPro program [5]. The intensities were corrected for Lorentz and polarization effects, and an absorption correction based on the multi-scan method using SCALE3 ABSPACK in CrysAlisPro was applied [5]. Data collection and refinement parameters are given in Table S3.

The structure was solved by direct methods using SHELXT [7], which revealed the positions of all non-hydrogen atoms of the title compound. The non-hydrogen atoms were refined anisotropically. All H-atoms were placed in geometrically calculated positions and refined

using a riding model where each H-atom was assigned a fixed isotropic displacement parameter with a value equal to 1.2Ueq of its parent atom.

Refinement of the structure was carried out on F^2 using full-matrix least-squares procedures, which minimized the function $\Sigma w(F_o^2 - F_c^2)^2$. The weighting scheme was based on counting statistics and included a factor to downweight the intense reflections. All calculations were performed using the SHELXL-2014/7 [8] program.

The crystals contains two molecules in the asymmetric unit (8 in the unit cell). Both molecules have the same chirality and they differ only in the conformation especially around the C-O bond of the C-O-$SiMe_3$. A careful check anyway revealed that there is not symmetry operation relating the two molecules and the unit cell and space group are correct.

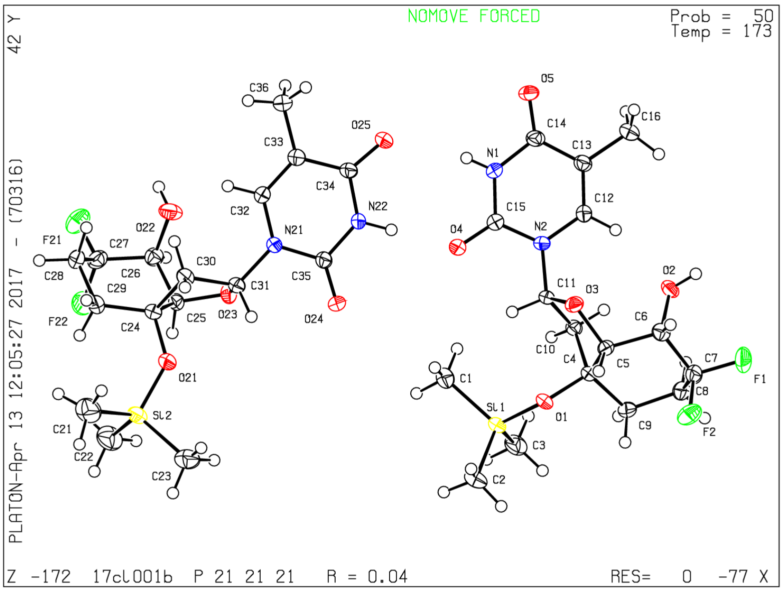

Figure S28: ORTEP plot of compound **6** with labeling.

Table S5: Crystal data and structure refinement for **6.**

Identification code	shelx	
Empirical formula	C16 H24 F2 N2 O5 Si	
Formula weight	390.46	
Temperature	173(2) K	
Wavelength	0.71073 Å	
Crystal system	Orthorhombic	
Space group	$P\ 2_12_12_1$	
Unit cell dimensions	*a* = 6.37620(10) Å	α= 90°.
	b = 15.4129(4) Å	β= 90°.
	c = 38.5411(6) Å	γ = 90°.
Volume	3787.65(13) $Å^3$	
Z	8	
Density (calculated)	1.369 Mg/m^3	
Absorption coefficient	0.172 mm^{-1}	
F(000)	1648	
Crystal size	0.2617 x 0.2035 x 0.0724 mm^3	
Theta range for data collection	1.692 to 27.331°.	
Index ranges	-7<=h<=7, -19<=k<=19, -48<=l<=49	
Reflections collected	28962	
Independent reflections	7769 [R(int) = 0.0335]	
Completeness to theta = 25.000°	99.9 %	
Absorption correction	Gaussian	
Max. and min. transmission	0.988 and 0.964	
Refinement method	Full-matrix least-squares on F^2	
Data / restraints / parameters	7769 / 0 / 487	
Goodness-of-fit on F^2	1.055	
Final R indices [I>2sigma(I)]	R1 = 0.0359, wR2 = 0.0728	
R indices (all data)	R1 = 0.0411, wR2 = 0.0749	
Absolute structure parameter	0.04(4)	
Extinction coefficient	n/a	
Largest diff. peak and hole	0.269 and -0.241 e.$Å^{-3}$	

Table S6: Atomic coordinates (x 10^4) and equivalent isotropic displacement parameters ($Å^2$x 10^3) for **6**. U(eq) is defined as one third of the trace of the orthogonalized U^{ij} tensor.

	x	y	z	U(eq)
C(1)	922(5)	6740(2)	5942(1)	33(1)
C(2)	739(6)	8152(2)	5394(1)	40(1)
C(3)	-3180(5)	7108(2)	5577(1)	37(1)
C(4)	-2789(4)	8532(2)	6282(1)	19(1)
C(5)	-1842(4)	9188(2)	6541(1)	19(1)
C(6)	-3401(4)	9715(2)	6755(1)	23(1)
C(7)	-5139(4)	10045(2)	6523(1)	27(1)
C(8)	-6271(4)	9351(2)	6330(1)	27(1)
C(9)	-4756(4)	8852(2)	6098(1)	23(1)
C(10)	-3012(4)	7735(2)	6515(1)	20(1)
C(11)	-931(4)	7782(2)	6705(1)	21(1)
C(12)	-2156(4)	7519(2)	7305(1)	22(1)
C(13)	-1919(4)	7228(2)	7629(1)	24(1)
C(14)	-101(4)	6707(2)	7707(1)	24(1)
C(15)	914(5)	6808(2)	7092(1)	36(1)
C(16)	-3417(5)	7456(2)	7916(1)	38(1)
C(21)	14619(5)	4455(3)	5150(1)	46(1)
C(22)	10804(6)	3456(2)	4883(1)	46(1)
C(23)	10562(6)	5396(2)	4994(1)	44(1)
C(24)	11044(4)	3696(2)	5834(1)	22(1)
C(29)	12904(4)	3091(2)	5773(1)	26(1)
C(28)	12877(4)	2263(2)	5993(1)	28(1)
C(27)	10787(5)	1835(2)	5971(1)	29(1)
C(26)	9010(4)	2393(2)	6099(1)	25(1)
C(25)	8920(4)	3227(2)	5882(1)	22(1)
C(30)	11115(4)	4253(2)	6162(1)	23(1)
C(31)	8841(4)	4541(2)	6182(1)	22(1)
C(32)	8715(4)	4439(2)	6831(1)	29(1)
C(33)	7847(5)	4606(2)	7143(1)	29(1)
C(34)	6014(4)	5145(2)	7153(1)	26(1)
C(35)	6287(4)	5328(2)	6518(1)	23(1)
C(36)	8701(5)	4250(2)	7477(1)	46(1)
N(1)	1141(4)	6503(2)	7423(1)	32(1)
N(2)	-802(4)	7323(1)	7037(1)	24(1)
N(21)	8013(3)	4785(1)	6524(1)	22(1)
N(22)	5409(4)	5487(2)	6839(1)	27(1)
O(2)	-4325(3)	9200(1)	7019(1)	27(1)
O(3)	-545(3)	8680(1)	6768(1)	22(1)
O(4)	2167(4)	6636(2)	6862(1)	70(1)
O(5)	434(3)	6449(1)	7995(1)	33(1)

O(1)	-1179(3)	8387(1)	6031(1)	23(1)
O(21)	10787(3)	4293(1)	5554(1)	29(1)
O(22)	9348(3)	2612(1)	6451(1)	32(1)
O(23)	7597(3)	3846(1)	6050(1)	25(1)
O(24)	5577(3)	5646(1)	6255(1)	29(1)
O(25)	4982(3)	5320(1)	7417(1)	35(1)
F(1)	-6509(3)	10524(1)	6718(1)	42(1)
F(2)	-4253(3)	10631(1)	6292(1)	38(1)
F(21)	10780(3)	1067(1)	6149(1)	43(1)
F(22)	10374(3)	1608(1)	5630(1)	39(1)
SI1	-733(1)	7605(1)	5746(1)	25(1)
SI2	11712(1)	4374(1)	5158(1)	26(1)

Table S7: Bond lengths [Å] and angles [°] **6**.

C(1)-SI1	1.862(3)
C(1)-H(1A)	0.9800
C(1)-H(1B)	0.9800
C(1)-H(1C)	0.9800
C(2)-SI1	1.851(3)
C(2)-H(2A)	0.9800
C(2)-H(2B)	0.9800
C(2)-H(2C)	0.9800
C(3)-SI1	1.855(3)
C(3)-H(3A)	0.9800
C(3)-H(3B)	0.9800
C(3)-H(3C)	0.9800
C(4)-O(1)	1.431(3)
C(4)-C(9)	1.524(3)
C(4)-C(10)	1.527(3)
C(4)-C(5)	1.543(3)
C(5)-O(3)	1.435(3)
C(5)-C(6)	1.526(3)
C(5)-H(5)	1.0000
C(6)-O(2)	1.417(3)
C(6)-C(7)	1.512(4)
C(6)-H(6)	1.0000
C(7)-F(1)	1.368(3)
C(7)-F(2)	1.389(3)
C(7)-C(8)	1.491(4)
C(8)-C(9)	1.525(4)
C(8)-H(8A)	0.9900
C(8)-H(8B)	0.9900
C(9)-H(9A)	0.9900

C(9)-H(9B)	0.9900
C(10)-C(11)	1.518(4)
C(10)-H(10A)	0.9900
C(10)-H(10B)	0.9900
C(11)-O(3)	1.426(3)
C(11)-N(2)	1.464(3)
C(11)-H(11)	1.0000
C(12)-C(13)	1.337(3)
C(12)-N(2)	1.379(3)
C(12)-H(12)	0.9500
C(13)-C(14)	1.442(4)
C(13)-C(16)	1.503(4)
C(14)-O(5)	1.227(3)
C(14)-N(1)	1.389(3)
C(15)-O(4)	1.222(3)
C(15)-N(1)	1.367(3)
C(15)-N(2)	1.368(4)
C(16)-H(16A)	0.9800
C(16)-H(16B)	0.9800
C(16)-H(16C)	0.9800
C(21)-SI2	1.858(3)
C(21)-H(21A)	0.9800
C(21)-H(21B)	0.9800
C(21)-H(21C)	0.9800
C(22)-SI2	1.858(3)
C(22)-H(22A)	0.9800
C(22)-H(22B)	0.9800
C(22)-H(22C)	0.9800
C(23)-SI2	1.850(3)
C(23)-H(23A)	0.9800
C(23)-H(23B)	0.9800
C(23)-H(23C)	0.9800
C(24)-O(21)	1.428(3)
C(24)-C(29)	1.527(4)
C(24)-C(30)	1.529(3)
C(24)-C(25)	1.546(4)
C(29)-C(28)	1.533(4)
C(29)-H(29A)	0.9900
C(29)-H(29B)	0.9900
C(28)-C(27)	1.489(4)
C(28)-H(28B)	0.9900
C(28)-H(28A)	0.9900
C(27)-F(21)	1.369(3)
C(27)-F(22)	1.387(3)
C(27)-C(26)	1.505(4)
C(26)-O(22)	1.418(3)

C(26)-C(25)	1.533(4)
C(26)-H(26)	1.0000
C(25)-O(23)	1.428(3)
C(25)-H(25)	1.0000
C(30)-C(31)	1.519(4)
C(30)-H(30A)	0.9900
C(30)-H(30B)	0.9900
C(31)-O(23)	1.426(3)
C(31)-N(21)	1.470(3)
C(31)-H(31)	1.0000
C(32)-C(33)	1.349(4)
C(32)-N(21)	1.372(3)
C(32)-H(32)	0.9500
C(33)-C(34)	1.435(4)
C(33)-C(36)	1.501(4)
C(34)-O(25)	1.241(3)
C(34)-N(22)	1.375(3)
C(35)-O(24)	1.212(3)
C(35)-N(22)	1.380(3)
C(35)-N(21)	1.383(3)
C(36)-H(36A)	0.9800
C(36)-H(36B)	0.9800
C(36)-H(36C)	0.9800
N(1)-H(1D)	0.84(3)
N(22)-H(22D)	0.91(3)
O(2)-H(2)	0.8400
O(1)-SI1	1.6549(18)
O(21)-SI2	1.6424(17)
O(22)-H(22)	0.8400
SI1-C(1)-H(1A)	109.5
SI1-C(1)-H(1B)	109.5
H(1A)-C(1)-H(1B)	109.5
SI1-C(1)-H(1C)	109.5
H(1A)-C(1)-H(1C)	109.5
H(1B)-C(1)-H(1C)	109.5
SI1-C(2)-H(2A)	109.5
SI1-C(2)-H(2B)	109.5
H(2A)-C(2)-H(2B)	109.5
SI1-C(2)-H(2C)	109.5
H(2A)-C(2)-H(2C)	109.5
H(2B)-C(2)-H(2C)	109.5
SI1-C(3)-H(3A)	109.5
SI1-C(3)-H(3B)	109.5
H(3A)-C(3)-H(3B)	109.5
SI1-C(3)-H(3C)	109.5
H(3A)-C(3)-H(3C)	109.5

H(3B)-C(3)-H(3C)	109.5
O(1)-C(4)-C(9)	108.91(18)
O(1)-C(4)-C(10)	109.8(2)
C(9)-C(4)-C(10)	117.2(2)
O(1)-C(4)-C(5)	105.1(2)
C(9)-C(4)-C(5)	114.3(2)
C(10)-C(4)-C(5)	100.67(19)
O(3)-C(5)-C(6)	109.69(19)
O(3)-C(5)-C(4)	105.09(19)
C(6)-C(5)-C(4)	116.3(2)
O(3)-C(5)-H(5)	108.5
C(6)-C(5)-H(5)	108.5
C(4)-C(5)-H(5)	108.5
O(2)-C(6)-C(7)	107.9(2)
O(2)-C(6)-C(5)	111.1(2)
C(7)-C(6)-C(5)	109.7(2)
O(2)-C(6)-H(6)	109.4
C(7)-C(6)-H(6)	109.4
C(5)-C(6)-H(6)	109.4
F(1)-C(7)-F(2)	105.2(2)
F(1)-C(7)-C(8)	110.6(2)
F(2)-C(7)-C(8)	110.1(2)
F(1)-C(7)-C(6)	109.0(2)
F(2)-C(7)-C(6)	107.5(2)
C(8)-C(7)-C(6)	114.1(2)
C(7)-C(8)-C(9)	110.4(2)
C(7)-C(8)-H(8A)	109.6
C(9)-C(8)-H(8A)	109.6
C(7)-C(8)-H(8B)	109.6
C(9)-C(8)-H(8B)	109.6
H(8A)-C(8)-H(8B)	108.1
C(4)-C(9)-C(8)	114.2(2)
C(4)-C(9)-H(9A)	108.7
C(8)-C(9)-H(9A)	108.7
C(4)-C(9)-H(9B)	108.7
C(8)-C(9)-H(9B)	108.7
H(9A)-C(9)-H(9B)	107.6
C(11)-C(10)-C(4)	99.4(2)
C(11)-C(10)-H(10A)	111.9
C(4)-C(10)-H(10A)	111.9
C(11)-C(10)-H(10B)	111.9
C(4)-C(10)-H(10B)	111.9
H(10A)-C(10)-H(10B)	109.6
O(3)-C(11)-N(2)	108.24(19)
O(3)-C(11)-C(10)	106.2(2)
N(2)-C(11)-C(10)	116.6(2)

O(3)-C(11)-H(11)	108.5
N(2)-C(11)-H(11)	108.5
C(10)-C(11)-H(11)	108.5
C(13)-C(12)-N(2)	123.7(2)
C(13)-C(12)-H(12)	118.2
N(2)-C(12)-H(12)	118.2
C(12)-C(13)-C(14)	118.3(2)
C(12)-C(13)-C(16)	122.5(3)
C(14)-C(13)-C(16)	119.1(2)
O(5)-C(14)-N(1)	118.9(2)
O(5)-C(14)-C(13)	126.4(2)
N(1)-C(14)-C(13)	114.7(2)
O(4)-C(15)-N(1)	122.1(3)
O(4)-C(15)-N(2)	122.6(2)
N(1)-C(15)-N(2)	115.3(2)
C(13)-C(16)-H(16A)	109.5
C(13)-C(16)-H(16B)	109.5
H(16A)-C(16)-H(16B)	109.5
C(13)-C(16)-H(16C)	109.5
H(16A)-C(16)-H(16C)	109.5
H(16B)-C(16)-H(16C)	109.5
SI2-C(21)-H(21A)	109.5
SI2-C(21)-H(21B)	109.5
H(21A)-C(21)-H(21B)	109.5
SI2-C(21)-H(21C)	109.5
H(21A)-C(21)-H(21C)	109.5
H(21B)-C(21)-H(21C)	109.5
SI2-C(22)-H(22A)	109.5
SI2-C(22)-H(22B)	109.5
H(22A)-C(22)-H(22B)	109.5
SI2-C(22)-H(22C)	109.5
H(22A)-C(22)-H(22C)	109.5
H(22B)-C(22)-H(22C)	109.5
SI2-C(23)-H(23A)	109.5
SI2-C(23)-H(23B)	109.5
H(23A)-C(23)-H(23B)	109.5
SI2-C(23)-H(23C)	109.5
H(23A)-C(23)-H(23C)	109.5
H(23B)-C(23)-H(23C)	109.5
O(21)-C(24)-C(29)	111.4(2)
O(21)-C(24)-C(30)	105.5(2)
C(29)-C(24)-C(30)	116.6(2)
O(21)-C(24)-C(25)	107.0(2)
C(29)-C(24)-C(25)	114.4(2)
C(30)-C(24)-C(25)	100.93(19)
C(24)-C(29)-C(28)	114.5(2)

C(24)-C(29)-H(29A)	108.6
C(28)-C(29)-H(29A)	108.6
C(24)-C(29)-H(29B)	108.6
C(28)-C(29)-H(29B)	108.6
H(29A)-C(29)-H(29B)	107.6
C(27)-C(28)-C(29)	110.3(2)
C(27)-C(28)-H(28B)	109.6
C(29)-C(28)-H(28B)	109.6
C(27)-C(28)-H(28A)	109.6
C(29)-C(28)-H(28A)	109.6
H(28B)-C(28)-H(28A)	108.1
F(21)-C(27)-F(22)	104.9(2)
F(21)-C(27)-C(28)	110.9(2)
F(22)-C(27)-C(28)	109.6(2)
F(21)-C(27)-C(26)	109.1(2)
F(22)-C(27)-C(26)	108.1(2)
C(28)-C(27)-C(26)	113.8(2)
O(22)-C(26)-C(27)	109.5(2)
O(22)-C(26)-C(25)	109.1(2)
C(27)-C(26)-C(25)	109.2(2)
O(22)-C(26)-H(26)	109.7
C(27)-C(26)-H(26)	109.7
C(25)-C(26)-H(26)	109.7
O(23)-C(25)-C(26)	109.7(2)
O(23)-C(25)-C(24)	105.0(2)
C(26)-C(25)-C(24)	115.1(2)
O(23)-C(25)-H(25)	108.9
C(26)-C(25)-H(25)	108.9
C(24)-C(25)-H(25)	108.9
C(31)-C(30)-C(24)	100.2(2)
C(31)-C(30)-H(30A)	111.7
C(24)-C(30)-H(30A)	111.7
C(31)-C(30)-H(30B)	111.7
C(24)-C(30)-H(30B)	111.7
H(30A)-C(30)-H(30B)	109.5
O(23)-C(31)-N(21)	108.2(2)
O(23)-C(31)-C(30)	107.1(2)
N(21)-C(31)-C(30)	117.5(2)
O(23)-C(31)-H(31)	107.9
N(21)-C(31)-H(31)	107.9
C(30)-C(31)-H(31)	107.9
C(33)-C(32)-N(21)	124.1(2)
C(33)-C(32)-H(32)	117.9
N(21)-C(32)-H(32)	117.9
C(32)-C(33)-C(34)	117.9(2)
C(32)-C(33)-C(36)	123.1(3)

C(34)-C(33)-C(36)	119.0(2)
O(25)-C(34)-N(22)	119.3(2)
O(25)-C(34)-C(33)	125.4(2)
N(22)-C(34)-C(33)	115.3(2)
O(24)-C(35)-N(22)	121.6(2)
O(24)-C(35)-N(21)	123.9(2)
N(22)-C(35)-N(21)	114.5(2)
C(33)-C(36)-H(36A)	109.5
C(33)-C(36)-H(36B)	109.5
H(36A)-C(36)-H(36B)	109.5
C(33)-C(36)-H(36C)	109.5
H(36A)-C(36)-H(36C)	109.5
H(36B)-C(36)-H(36C)	109.5
C(15)-N(1)-C(14)	126.8(2)
C(15)-N(1)-H(1D)	115(2)
C(14)-N(1)-H(1D)	118(2)
C(15)-N(2)-C(12)	120.9(2)
C(15)-N(2)-C(11)	117.4(2)
C(12)-N(2)-C(11)	120.8(2)
C(32)-N(21)-C(35)	120.7(2)
C(32)-N(21)-C(31)	123.9(2)
C(35)-N(21)-C(31)	115.1(2)
C(34)-N(22)-C(35)	127.4(2)
C(34)-N(22)-H(22D)	116.0(19)
C(35)-N(22)-H(22D)	116.6(19)
C(6)-O(2)-H(2)	109.5
C(11)-O(3)-C(5)	109.18(18)
C(4)-O(1)-SI1	133.43(16)
C(24)-O(21)-SI2	135.36(16)
C(26)-O(22)-H(22)	109.5
C(31)-O(23)-C(25)	109.55(19)
O(1)-SI1-C(2)	103.94(13)
O(1)-SI1-C(3)	112.84(12)
C(2)-SI1-C(3)	111.01(15)
O(1)-SI1-C(1)	110.38(11)
C(2)-SI1-C(1)	109.69(15)
C(3)-SI1-C(1)	108.89(15)
O(21)-SI2-C(23)	103.93(12)
O(21)-SI2-C(21)	112.23(13)
C(23)-SI2-C(21)	109.45(18)
O(21)-SI2-C(22)	111.05(14)
C(23)-SI2-C(22)	109.27(16)
C(21)-SI2-C(22)	110.66(17)

Symmetry transformations used to generate equivalent atoms:

Table S8: Anisotropic displacement parameters ($Å^2$x 10^3) for **6**. The anisotropic displacement factor exponent takes the form: $-2\pi^2[h^2a^{*2}U^{11} + ... + 2 h k a^* b^* U^{12}]$

	U^{11}	U^{22}	U^{33}	U^{23}	U^{13}	U^{12}
C(1)	40(2)	32(2)	28(1)	-3(1)	5(1)	9(2)
C(2)	48(2)	51(2)	22(1)	7(1)	11(1)	7(2)
C(3)	41(2)	47(2)	23(1)	-9(1)	-2(1)	3(2)
C(4)	20(1)	21(1)	15(1)	1(1)	2(1)	2(1)
C(5)	19(1)	20(1)	19(1)	2(1)	-1(1)	1(1)
C(6)	28(1)	18(1)	22(1)	-2(1)	1(1)	-2(1)
C(7)	32(2)	22(1)	27(1)	2(1)	6(1)	7(1)
C(8)	21(1)	33(2)	26(1)	3(1)	1(1)	4(1)
C(9)	25(1)	27(2)	18(1)	1(1)	-4(1)	2(1)
C(10)	25(1)	19(1)	18(1)	0(1)	1(1)	2(1)
C(11)	27(1)	23(1)	14(1)	2(1)	2(1)	7(1)
C(12)	23(1)	21(1)	22(1)	0(1)	2(1)	4(1)
C(13)	31(1)	20(1)	19(1)	1(1)	4(1)	1(1)
C(14)	36(2)	19(1)	18(1)	0(1)	0(1)	2(1)
C(15)	43(2)	42(2)	21(1)	6(1)	4(1)	22(2)
C(16)	45(2)	46(2)	23(1)	8(1)	11(1)	14(2)
C(21)	34(2)	59(2)	45(2)	7(2)	9(2)	-7(2)
C(22)	53(2)	50(2)	35(2)	-13(2)	-4(2)	-1(2)
C(23)	57(2)	45(2)	31(2)	10(1)	6(2)	4(2)
C(24)	24(1)	24(1)	17(1)	1(1)	1(1)	2(1)
C(29)	23(1)	30(2)	24(1)	-2(1)	3(1)	4(1)
C(28)	27(1)	30(2)	26(1)	0(1)	-2(1)	10(1)
C(27)	37(2)	22(1)	27(1)	1(1)	-5(1)	5(1)
C(26)	24(1)	26(1)	25(1)	-1(1)	-2(1)	0(1)
C(25)	22(1)	25(1)	19(1)	-4(1)	-1(1)	5(1)
C(30)	24(1)	24(1)	20(1)	-1(1)	3(1)	0(1)
C(31)	25(1)	23(1)	17(1)	-1(1)	1(1)	2(1)
C(32)	33(2)	31(2)	21(1)	2(1)	1(1)	14(1)
C(33)	37(2)	29(2)	21(1)	2(1)	3(1)	10(1)
C(34)	34(2)	24(1)	18(1)	4(1)	5(1)	6(1)
C(35)	28(1)	19(1)	21(1)	1(1)	3(1)	4(1)
C(36)	55(2)	63(2)	22(1)	9(2)	4(1)	33(2)
N(1)	39(2)	39(2)	19(1)	6(1)	2(1)	21(1)
N(2)	33(1)	26(1)	15(1)	3(1)	2(1)	9(1)
N(21)	27(1)	23(1)	17(1)	1(1)	3(1)	7(1)
N(22)	32(1)	30(1)	19(1)	5(1)	6(1)	16(1)
O(2)	37(1)	25(1)	19(1)	-2(1)	8(1)	5(1)
O(3)	23(1)	25(1)	19(1)	1(1)	-4(1)	2(1)
O(4)	79(2)	106(2)	24(1)	24(1)	20(1)	71(2)
O(5)	49(1)	32(1)	17(1)	4(1)	-2(1)	13(1)

O(1)	26(1)	28(1)	15(1)	0(1)	5(1)	4(1)
O(21)	35(1)	30(1)	21(1)	5(1)	8(1)	8(1)
O(22)	48(1)	30(1)	20(1)	4(1)	2(1)	-2(1)
O(23)	21(1)	27(1)	28(1)	-4(1)	2(1)	4(1)
O(24)	34(1)	34(1)	19(1)	6(1)	0(1)	13(1)
O(25)	45(1)	40(1)	20(1)	7(1)	10(1)	17(1)
F(1)	43(1)	40(1)	44(1)	-11(1)	3(1)	20(1)
F(2)	46(1)	26(1)	42(1)	14(1)	-3(1)	1(1)
F(21)	50(1)	25(1)	54(1)	10(1)	-4(1)	4(1)
F(22)	47(1)	36(1)	34(1)	-14(1)	-9(1)	7(1)
SI1	30(1)	30(1)	15(1)	-1(1)	3(1)	6(1)
SI2	30(1)	30(1)	19(1)	0(1)	5(1)	-1(1)

6. References

1. Stauffiger, A.; Leumann, C. J. *Eur. J. Org. Chem.* **2009**, *2009*, 1153–1162.
2. *Gaussian 09*, Revision A.02; Gaussian, Inc.: Wallingford, CT, 2009.
3. *OriginPro*; OriginLab: Northampton, MA, 2016.
4. Frei, S.; Leumann, C. J. *Beilstein J. Org. Chem.,* in press.
5. *CrysAlisPro,* Version 1.171.34.44; Oxford Diffraction Ltd: Yarnton, Oxfordshire, UK, 2010.
6. Macchi, P.; Bürgi, H.B.; Chimpri, A. S.; Hauser, J.; Gál, Z. *J. Appl. Cryst.* **2011**, *44*, 763–771.
7. Sheldrick, G. M. *Acta Cryst.* **2015**, *A71*, 3–8.
8. Sheldrick, G. M.. *Acta Cryst.* **2015**, C*71*, 3–8.
